# A tumor immune microenvironment gene expression signature for predicting prognosis, immunotherapy efficacy, and drug candidates in triple-negative breast cancer

**DOI:** 10.3389/fimmu.2025.1676768

**Published:** 2025-11-10

**Authors:** Li Bai, Ziyu Zhou, Qingqing Yu, Zhen Ye, Qingzhou Li, Shengrong Li, Congcong Li, Yu Hu, Yunjie Hu, Xinran Tai, Lei Xiang, Sijuan Sun, Jianya Deng, Yumei Wang, Dong Wang

**Affiliations:** 1 School of Pharmacy, State Key Laboratory of Southwestern Chinese Medicine Resources, Chengdu University of Traditional Chinese Medicine, Chengdu, China; 2 School of Basic Medical Sciences, Chengdu University of Traditional Chinese Medicine, Chengdu, China

**Keywords:** tumor immune microenvironment, immunotherapy, gene expression signature, nitidine chloride, JAK-STAT signaling pathway

## Abstract

**Background:**

Immunotherapy has transformed cancer treatment, but its efficacy remains limited in patients with immunologically “cold” tumors. Triple-negative breast cancer (TNBC), despite elevated PD-L1 expression and high tumor mutation burden, often exhibits poor T cell infiltration, rendering it largely unresponsive to immune checkpoint blockade. Overcoming the immunosuppressive tumor immune microenvironment (TIME) remains a major challenge in oncology.

**Methods:**

We defined a tumor immune microenvironment gene expression signature (TIME-GES) through transcriptomic analysis of clinical samples. Its performance and relevance were evaluated using representative approaches including enrichment analysis, immune infiltration profiling, receiver operating characteristic analysis, and survival assessment. Based on TIME-GES, we screened 1,865 natural compounds and identified Nitidine Chloride (NCD) as a potential modulator in TNBC. *In vivo* efficacy of NCD against TNBC was examined by representative assays such as flow cytometry and immunofluorescence. Mechanistic insights into TNBC treatment via TIME-GES were explored through RNA sequencing, quantitative PCR, Western blotting, and cellular thermal shift assay.

**Results:**

TIME-GES effectively characterizes the tumor immune microenvironment across diverse cancer types. It reliably distinguishes tumor immune phenotypes and predicts patient responses to immunotherapy. Moreover, TIME-GES is strongly associated with survival outcomes in patients receiving immunotherapy and remains a significant prognostic marker for overall survival and mortality in TCGA pan-cancer cohorts, regardless of treatment. Guided by TIME-GES, NCD was identified from a natural product library and shown to modulate TIME-GES gene expression and significantly inhibit TNBC growth *in vivo*. NCD enhances CD8^+^ T cell–mediated antitumor immunity by upregulating TIME-GES genes and targeting the JAK2-STAT3 signaling pathway, resulting in suppressed tumor growth and reprogramming of the TIME toward a more immunologically active, “hot” phenotype.

**Conclusion:**

This study identified TIME-GES as a novel biomarker capable of distinguishing tumor immune phenotypes, predicting immunotherapy response, and evaluating prognosis in TNBC. Furthermore, TIME-GES-guided screening led to the discovery of NCD, a promising immunomodulatory agent that reprograms the TIME and enhances anti-tumor immunity in TNBC. This study offers both a robust immune gene signature and a candidate therapeutic to improve immunotherapy outcomes in TNBC.

## Graphical Abstract

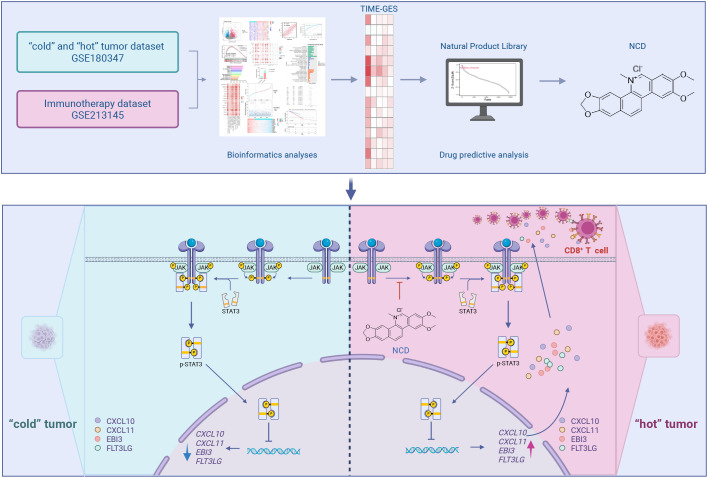

## 1 Introduction

Tumor immunotherapy, by activating the immune system to precisely target tumor cells, has overcome the limitations of traditional chemotherapy and radiotherapy (1), significantly extending patients’ survival (2). Despite its breakthrough efficacy in various cancers, the response rate among patients varies considerably, largely due to the high heterogeneity of the tumor immune microenvironment (TIME). Studies have demonstrated that the degree of immune cell infiltration within the TIME is a key determinant of immunotherapy efficacy: “hot” tumors, characterized by high tumor-infiltrating lymphocytes (TILs), are highly responsive to immunotherapy and associated with better prognosis (3), whereas “cold” tumors, with low TILs infiltration, respond poorly to treatment and have worse outcomes (4). The overall immune landscape of a tumor is shaped by the balance between pro-tumor and anti-tumor immune activities, which collectively determine its immunological phenotype (5). The fundamental distinction between these two phenotypes lies in the ability of T cells to effectively infiltrate tumor tissues. Therefore, enhancing T cell migration into the tumor microenvironment is a critical strategy for converting “cold” tumors into “hot” tumors (3), thereby improving the efficacy of immunotherapy.

Triple-negative breast cancer (TNBC) is the most aggressive subtype of breast cancer (BC). Due to the lack of estrogen receptor, progesterone receptor, and human epidermal growth factor receptor 2 in TNBC cells, patients do not benefit from endocrine or targeted therapies. Although chemotherapy and radiotherapy remain the main treatment options for TNBC, its aggressive nature, early metastasis (6), and high recurrence rate (7) limit clinical efficacy, necessitating improved patient survival strategies. TNBC is characterized by high PD-L1 expression and tumor mutation burden levels (8), making it more suitable for immunotherapy compared to traditional therapies. However, its typical “cold” tumor phenotype (9) leads to poor immunotherapy outcomes. Therefore, reversing the “cold” tumor phenotype of TNBC through pharmacological means to enhance immunotherapy efficacy represents a therapeutic breakthrough.

Physiological and pathological states are characterized by distinct phenotypic and gene expression profiles. Compared to traditional phenotypic analyses, transcriptional alterations provide deeper insights into the biological mechanisms underlying disease and can inform multiple aspects of clinical decision-making, including treatment strategies, drug development, prognostic evaluation, and diagnostic assessment (10–12). Gene expression signatures represent the expression patterns of cells or tissues under specific conditions, linking diseases, genes, and drugs (13). Currently, high-throughput sequencing-based high-throughput screening (HTS^2^) and other similar technologies, including highly multiplexed and parallel sequencing (HiMAP-seq) (14), which could detect the expression of thousands of genes in thousands of samples per test, have been successfully applied in gene signature guided drug screening (15–18). By leveraging the gene expression signatures of “cold” and “hot” tumors, combined with drug perturbed gene expression database, we can perform large-scale screening for drugs with immune microenvironment regulatory potential.

As illustrated in the graphical abstract, this study leverages tumor transcriptomic datasets to define a gene signature representative of TIME. This signature is then integrated with drug induced transcriptome datasets to systematically identify candidate drugs with potential immunotherapeutic efficacy for the treatment of TNBC. First, we established a TIME gene expression signature (TIME-GES) by analyzing datasets of “cold” and “hot” tumors and immunotherapy-treated samples. Subsequently, we systematically evaluated the performance of TIME-GES in distinguishing tumor immune phenotypes, predicting immunotherapy response, and evaluating patient prognosis through immune infiltration analysis, survival analysis, and other bioinformatics approaches. Finally, based on TIME-GES, we screened a transcriptome dataset containing 1,865 natural compounds and identified a potential drug, Nitidine Chloride (NCD). Through *in vitro* and *in vivo* experiments, we disclosed that NCD upregulates the TIME-GES genes *CXCL10*, *CXCL11*, *EBI3*, and *FLT3LG*, promotes CD8^+^ T cell activation and tumor infiltration, thereby inhibiting the growth of TNBC.

## 2 Methods

### 2.1 TIME-GES

To construct the TIME-GES gene set, differential expression analyses were applied to lung adenocarcinoma datasets (19) using the limma package and to anti-PD-1-treated melanoma datasets (20) using DESeq2. Genes with |log_2_FC| > 1 and *P* < 0.05 were selected. The intersection of consistently up- or downregulated genes across both datasets yielded a signature. According to prior studies (21), each gene was assigned a score of “+1” or “-1” depending on whether its expression was elevated or reduced in “hot” tumors, and the aggregated score defined the TIME-GES score.

### 2.2 Enrichment analysis

Gene Set Enrichment Analysis (GSEA) was carried out using the clusterProfiler package in R. Differentially expressed genes (DEGs) were analyzed for pathway involvement through enrichKEGG and enrichGO functions, with *P* < 0.05 as the cutoff.

### 2.3 Correlation analysis for TIME-GES and immune

Gene expression data and patient clinical information for 30 cancers (including BC) were obtained from the UCSC Xena database (UCSC Xena, https://xena.ucsc.edu/) (22), combined with TILs abundance data from TISIDB (TISIDB, http://cis.hku.hk/TISIDB/) (23). Correlations between TIME-GES genes and 14 immune cell types were analyzed using R’s “cor.test” function. The “Immune Estimation” module of the TIMER2.0 platform (TIMER2.0, http://timer.comp-genomics.org/) (24) was used to evaluate the correlation between TIME-GES genes and CD8^+^ T cell infiltration across 40 cancer types.

### 2.4 Evaluation of TIME-GES for distinguishing tumor immune phenotypes

The ROCR package in R was employed to evaluate the predictive performance of TIME-GES for distinguishing “cold” and “hot” tumors in lung adenocarcinoma datasets (19), primarily by calculating the area under the curve (AUC). To enable a comparative analysis, six tumor immune-related gene signatures (25–30) and four established immune checkpoint blockade (ICB) response biomarkers were also included for AUC evaluation. In addition, for TIME-GES, precision, recall, and accuracy were further assessed using ROCR to comprehensively characterize its classification performance.

### 2.5 Clinical data analysis

The R survival package’s “coxph” function was used to analyze the associations between the expression of TIME-GES genes and overall survival (OS). Kaplan–Meier survival analyses were performed for OS, disease-specific survival (DSS), progression-free interval (PFI), and disease-free interval (DFI). Risk scores were derived from Cox regression coefficients, with patients stratified into high or low-risk groups. Immunotherapy datasets GSE181815 (31), GSE210287 (32), GSE91061 (33) and GSE93157 (34) (GEO, https://www.ncbi.nlm.nih.gov/geo/) (35) were processed using DESeq2. Kaplan–Meier survival analyses as well as univariate Cox regression analyses were performed for melanoma patients with GSE93157 (34) with Progression-Free Survival (PFS) information.

### 2.6 Drug screening

The drug screening data used in this program was constructed by the lab upfront, based on a database of 1,865 natural compounds perturbation generated using HiMAP-seq technology (14). Gene expression data for TIME-GES–associated genes in TNBC cell lines (MDA-MB-231, 21MT2, HCC1143, and HCC1806) were obtained from DepMap (DepMap, https://depmap.org/portal/) (36). A cutoff of average normalized gene expression >1 was applied to identify highly expressed genes (*CXCL10*, *CXCL11*, *EBI3* and *FLT3LG*). Subsequently, probe-based filtering refined the candidate gene set to *CXCL11*, *EBI3* and *FLT3LG*. To prioritize compounds, drug–gene expression profile similarity was evaluated using the RCSM R package.

### 2.7 Cell culture

MDA-MB-231 and 4T1 cell lines were sourced from the Chinese Cancer Cell Line Encyclopedia. MDA-MB-231 cells were grown in Dulbecco’s Modified Eagle Medium (DMEM, Gibco) supplemented with 10% fetal bovine serum (FBS, ExCell) and 1% penicillin–streptomycin solution (Hyclone). For 4T1 cells, Roswell Park Memorial Institute (RPMI)-1640 medium (Gibco) with 10% FBS and 1% penicillin–streptomycin was used. All cultures were maintained at 37°C in a humidified incubator with 5% CO_2_.

### 2.8 Cell proliferation

Cells were plated in 96-well plates at a density of 5 × 10^3^ cells per well. After 24 hours of incubation, the medium was replaced with fresh medium containing different concentrations of NCD (Push-Herbchem), which were dissolved in dimethyl sulfoxide (DMSO, Solarbio) and incubated for another 24 hours. DMSO-treated wells served as vehicle controls, while wells without cells were used as blanks. Cell viability was measured using the Cell Counting Kit-8 (CCK-8) assay (bgbiotech) following the manufacturer’s instructions.

### 2.9 Quantitative polymerase chain reaction

Total RNA was isolated with RNA isolater Total RNA Extraction Reagent (Vazyme), followed by reverse transcription using the RT Easy™ II cDNA Synthesis Kit (Foregene). Quantitative polymerase chain reaction (qPCR) was then carried out with gene-specific primers listed in **Supplementary Table S1**.

### 2.10 Animal experiments

Experiments were carried out in facilities accredited by AAALAC and were approved by the Institutional Animal Care and Use Committee of Chengdu University of Traditional Chinese Medicine (Approval ID: 20240106). The cages were arranged by the staffs of the laboratory animal research facility of Chengdu University of TCM, the staffs were not aware of the study design. An adaptable environment with a natural light cycle, room temperature (20–23°C), and 50%–60% humidity was provided for seven days prior to the experiment. A total of 2.5 × 10^4^ 4T1 cells in 20 μL of RPMI-1640 medium were orthotopically injected into the mammary fat pads of 6-week-old wild-type female BALB/c mice (3 mice per cage). The mice were arranged into groups using a random number method. Mice in the NCD treatment cohorts received daily intraperitoneal injections of 2.5 or 5 mg/kg, prepared in 5% DMSO/PBS, for 3 weeks. A positive control group was treated with cisplatin (1.5 mg/kg), also prepared in 5% DMSO/PBS, administered three times per week over 2 weeks. Two additional control groups were included as negative controls: “Control” consisted of non-tumor-bearing mice, and “Model” consisted of tumor-bearing mice that received vehicle only. Both groups were administered equivalent volumes of 5% DMSO/PBS intraperitoneally, consistent with the treatment groups. Body weight change was assessed daily. Tumor volume was set as the primary endpoint. Tumor dimensions were recorded weekly, and volumes were calculated using the formula: Tumor volume (mm³) = (Width² × Length)/2. At the end of the study, tumors and blood samples were collected for downstream analyses. The animals were sacrificed in the designated dissection facility.

### 2.11 Flow cytometry

For immunophenotyping, cells were labeled with the following fluorophore-conjugated antibodies: Brilliant Violet™ 421-conjugated anti-CD4, PerCP-Cy5.5-conjugated anti-CD8a, FITC-conjugated anti-CD3, and APC-eFluor 780-conjugated anti-CD45. Cell viability was assessed using Fixable Viability Dye eFluor™ 506. All reagents are from Invitrogen. Samples were analyzed using FlowJo software.

### 2.12 Biochemistry test

After blood collection from mice, the samples were left to stand at room temperature for 1 hour, followed by centrifugation at 1000 × g for 20 minutes at 4°C. The supernatant serum was carefully collected and subjected to biochemical analysis using an Auto Chemistry Analyzer BS-240 Vet (Mindray). Specific biochemical parameters, including aspartate aminotransferase (AST), alanine aminotransferase (ALT), albumin (ALB), creatinine (CREA), and UREA, were measured using corresponding assay kits (Mindray).

### 2.13 Immunofluorescence and hematoxylin–eosin staining

Tumor tissues and spleen tissues were fixed in 4% paraformaldehyde (Servicebio), embedded in paraffin, and sectioned at 4 μm thickness. For hematoxylin-eosin staining (H&E), tissue sections were stained with Hematoxylin (Sigma-Aldrich) and Eosin Y, free acid (Ruibio). For immunofluorescence (IF), sections underwent antigen retrieval and were incubated with Anti-CD8 alpha antibody (Abcam), followed by fluorescently labeled secondary antibodies. Nuclei were counterstained with DAPI (Servicebio).

### 2.14 RNA sequencing

A total of 2 × 10^5^ MDA-MB-231 cells were plated in 6-well dishes and exposed to 5 μM NCD for 24 hours. RNA was isolated using RNA isolater Total RNA Extraction Reagent following the manufacturer’s protocol. cDNA libraries were constructed using the Illumina NovaSeq Reagent Kit (Illumina) and subjected to high-throughput sequencing on the NovaSeq X Plus system. Sequencing reads were aligned to the human reference genome using HiSat2, and gene expression levels were quantified via RSEM. Differential gene expression analysis was performed using DESeq2, with cutoff criteria set at |log_2_FC| > 1 and *P* < 0.05.

### 2.15 Western blot analysis

Total protein was extracted from cells using RIPA lysis buffer (Beyotime) supplemented with broad spectrum protease inhibitor cocktail (Boster) and broad-spectrum phosphatase inhibitor cocktail (Boster). Protein concentrations were quantified using a BCA Protein Assay Kit (CWBio). Equal amounts of protein were then loaded onto SDS-PAGE gels prepared with the One-Step PAGE Gel Fast Preparation Kit (Vazyme), electrophoresed, and transferred to an Immobilon^®^-PSQ PVDF membrane (Millipore). Membranes were blocked and then incubated with the following primary antibodies: Stat3 (124H6) Mouse mAb, Phospho-Stat3 (Tyr705) (D3A7) XP^®^ Rabbit mAb, Jak2 (D2E12) XP^®^ Rabbit mAb, Phospho-Jak2 (Tyr1007/1008) Antibody (all from Cell Signaling Technology), and Alpha Tubulin Monoclonal antibody (Proteintech). Chemiluminescent signals were detected using the UltraSignal hypersensitive ECL chemiluminescence substrate (4A Biotech).

### 2.16 Plasmids

Lentiviral shRNAs were provided by the Vector Core at Tsinghua University, with specific sequences detailed in **Supplementary Table S2**.

### 2.17 Cellular thermal shift assay

Cells were treated with either 20 μM NCD or DMSO for 1.5 hours. Following treatment, three freeze-thaw cycles were applied to lyse the cells. The lysates were then incubated at a range of temperatures and centrifuged at 12,000 rpm for 20 minutes at 4°C. Supernatants were collected and mixed with Omni-Easy™ loading buffer (EpiZyme), heated at 95°C for 10 minutes, and subjected to Western blot analysis to evaluate protein stability.

### 2.18 Surface plasmon resonance

The binding interaction between NCD and JAK2 protein was evaluated using a Biacore 1K surface plasmon resonance (SPR) system (Cytiva). Recombinant human JAK2 protein (TargetMol) was immobilized on a CM5 sensor chip (Cytiva) through standard amine-coupling chemistry. Briefly, the sensor surface was activated with a mixture of 0.2 M EDC (Cytiva) and 0.05 M NHS (Cytiva), followed by injection of JAK2 at a concentration of 20 μg/mL in 10 mM sodium acetate buffer (pH 4.5) (Cytiva). Unreacted sites were blocked with 1 M ethanolamine (pH 8.5) (Cytiva). Regeneration of the sensor surface was achieved using 10 mM glycine-HCl (pH 2.5) (Cytiva). The compound was prepared in running buffer (PBS with 5% DMSO) at a series of concentrations (0.19, 0.39, 0.78, 1.56, 3.12, 6.25, 12.5 and 25.0 μM) and injected over the immobilized JAK2 surface at a flow rate of 30 μL/min at 25 °C. Each injection included an association phase of 90 s and a dissociation phase of 60 s. Binding responses were recorded in real-time, and the equilibrium dissociation constant (KD) were analyzed using Biacore Evaluation Software (GE Healthcare).

### 2.19 Statistical analysis

Image quantification was performed using ImageJ software. All data were processed using GraphPad Prism version 8.0. Two-tailed unpaired Student’s t-tests were applied for comparisons between two groups, while differences among multiple groups were assessed using one-way ANOVA. The statistical data were presented as “mean ± standard error”. Significance was indicated as follows: *P < 0.05, **P < 0.01, ***P < 0.001, ****P < 0.0001. Pearson’s correlation was used to examine relationships between variables.

## 3 Results

### 3.1 Construction of TIME-GES

**Figure 1:**
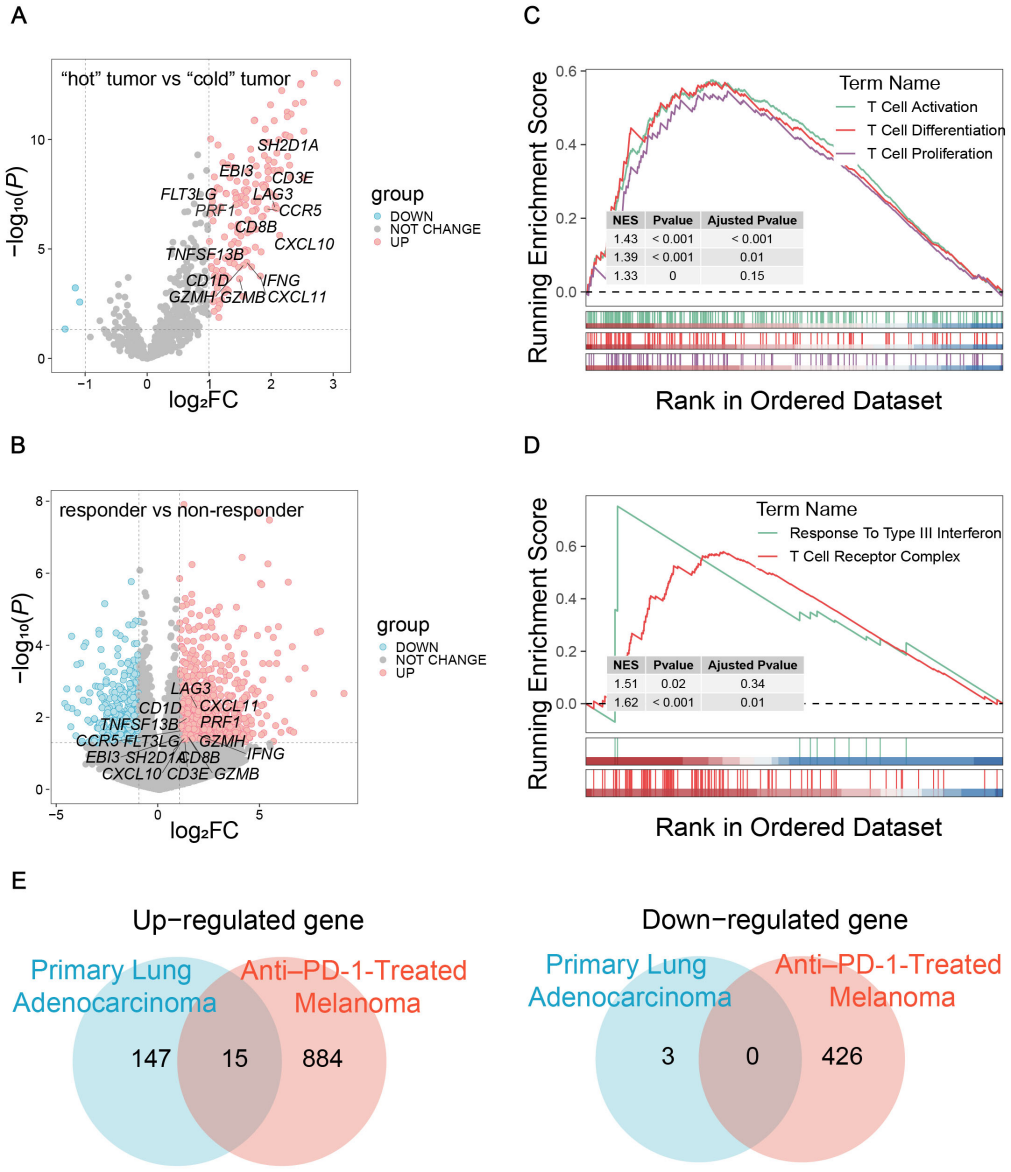
Construction of TIME-GES. **(A)** Volcano plots showed the DEGs in primary lung adenocarcinoma cohort. **(B)** Volcano plots showed the DEGs in anti–PD-1-treated melanoma cohort. **(C, D)** GSEA showed that immune related pathways enriched by DEGs in primary lung adenocarcinoma and anti–PD-1-treated melanoma cohorts, respectively. **(E)** Venn diagram showing overlapped up-regulated and down-regulated DEGs between primary lung adenocarcinoma and anti–PD-1-treated melanoma cohorts.

To establish a TIME-GES, we integrated transcriptomic data from two independent cohorts: primary lung adenocarcinoma (GSE180347, n=144) (19) and treatment-naïve anti-PD-1-treated melanoma (GSE213145, n=20) (20). In the lung adenocarcinoma cohort, patients were divided into “cold” (n=60) and “hot” (n=84) tumor groups based on histopathological lymphocyte infiltration levels (HPF) counts. In the melanoma cohort, patients were stratified into responders (n=10) and non-responder (n=10) groups according to their immunotherapy response. Based on the analyses, we respectively identified 165 and 1,325 DEGs in the lung adenocarcinoma and melanoma cohorts, respectively (**Figures 1A, B**). The GSEA analysis revealed significant enrichment of T cell-associated processes in both datasets (**Figures 1C, D**). We intersected the upregulated and downregulated genes from both cohorts separately and identified 15 overlapped genes exclusively among the upregulated set (*CCR5*, *CD1D*, *CD3E*, *CD8B*, *CXCL10*, *CXCL11*, *EBI3*, *FLT3LG*, *GZMB*, *GZMH*, *IFNG*, *LAG3*, *PRF1*, *SH2D1A* and *TNFSF13B*), which were collectively defined as the TIME-GES (**Figure 1E**). The TIME-GES illustrated a coordinated high-expression pattern in the “hot” tumor group and the treatment responder group (**Supplementary Figures S1A, B**). Furthermore, TIME-GES genes were significantly associated with immune-related pathways (**Supplementary Figures S1C, D**).

### 3.2 TIME-GES reflects tumor immune microenvironment characteristics

**Figure 2:**
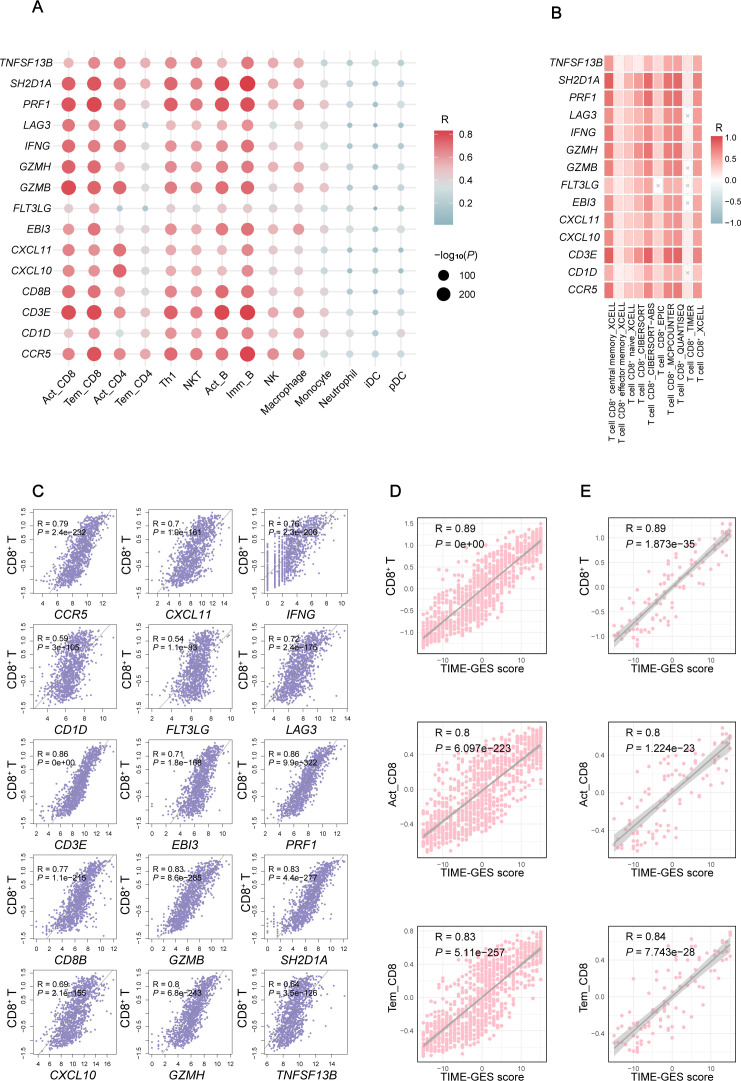
TIME-GES reflects TIME characteristics. **(A)** Correlation of TIME-GES genes expression with the infiltration for 14 immune cell types in BC. **(B)** Correlation of TIME-GES genes expression with T cells infiltration using multiple algorithms in BC. **(C)** Correlation between TIME-GES gene expression and CD8^+^ T cells in BC. **(D)** Correlation between TIME-GES score and CD8^+^ T, Act_CD8, and Tem_CD8 in BC. **(E)** Correlation between TIME-GES score and CD8^+^ T, Act_CD8 and Tem_CD8 cells in TNBC.

To explore the relationship between TIME-GES and tumor immune status, we performed correlation analyses between TIME-GES and immune cell infiltration in a BC cohort. The results showed that all 15 TIME-GES genes exhibited significant positive correlations (*P* < 0.05) with 14 immune cell subsets, including activated CD8^+^ T cell (Act_CD8), effector memory CD8^+^ T cell (Tem_CD8), activated CD4^+^ T cell (Act_CD4), effector memory CD4^+^ T cell (Tem_CD4), Type 1 T helper cell (Th1), natural killer T cell (NKT), activated B cell (Act_B), immature B cell (Imm_B), natural killer cell (NK), Macrophage, Monocyte, Neutrophil, immature dendritic cell (iDC) and plasmacytoid dendritic cell (pDC) (**Figure 2A**). Among these, T cell subsets (particularly Act_CD8 and Tem_CD8) displayed the strongest correlations with the TIME-GES (mean Pearson’s R > 0.65). And this correlation pattern was consistently observed across 29 additional tumor types (**Supplementary Figures S2**-**S5**).

To further elucidate the relationship between TIME-GES and CD8^+^ T cells infiltration, we employed multiple computational algorithms, including CIBERSORT, xCell, and TIMER, to assess the correlation in BC patients. The analysis revealed that nearly all 15 TIME-GES genes were significantly positively correlated with CD8^+^ T cells across different algorithms (**Figure 2B**). This correlation was also observed across 29 other tumor types, including adrenocortical carcinoma (ACC), bladder urothelial carcinoma (BLCA), Cervical squamous cell carcinoma and endocervical adenocarcinoma (CESC) (**Supplementary Figures S6**-**S8**). In addition, each TIME-GES gene exhibited significant positive correlations with CD8^+^ T cells (**Figure 2C**). What’s more, the TIME-GES composite score showed a strong positive correlation with Act_CD8, Tem_CD8, and overall CD8^+^ T cell infiltration levels in both BC (R > 0.8, *P* < 0.001) and TNBC (R > 0.8, *P* < 0.001) patients (**Figures 2D, E**). These findings underscore the strong association between TIME-GES and tumor immune status.

### 3.3 TIME-GES predicts responsiveness to cancer immunotherapy

**Figure 3:**
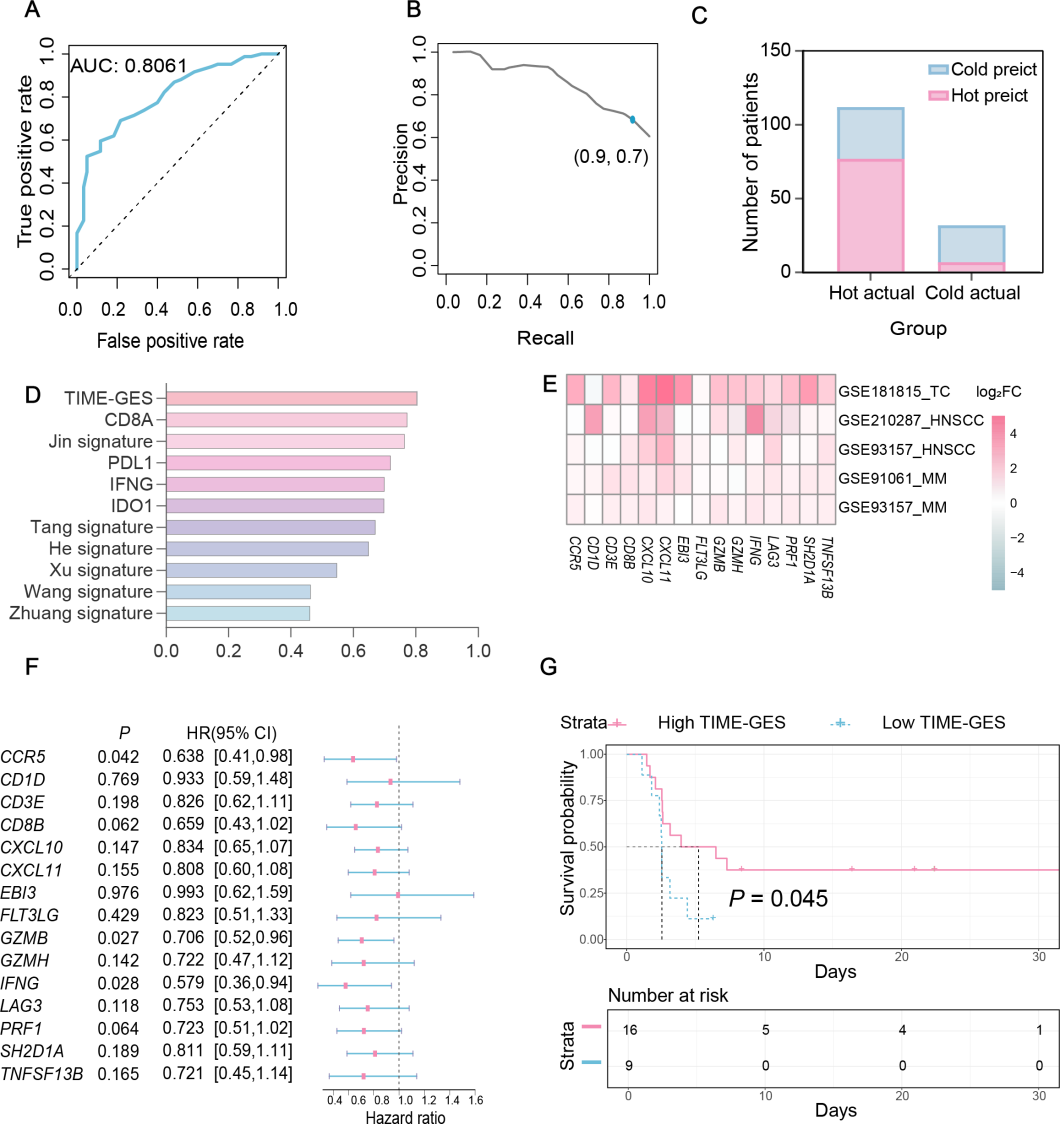
TIME-GES predicts responsiveness to cancer immunotherapy. **(A-C)** Performance metrics of TIME-GES in predicting “hot” and “cold” tumors in primary lung adenocarcinoma. **(D)** Comparison of AUC. values among TIME-GES, six immune gene panels, and four known ICB biomarkers. **(E)** Heatmap for log_2_FC of TIME-GES genes expression between responders and non-responders across five cohorts (Thymic carcinoma: TC, MM: Melanoma, HNSC: Head and Neck squamous cell carcinoma). Red indicates genes expressed at higher levels in responders, blue indicates lower levels, and white indicates no difference; color intensity reflects the magnitude of difference. **(F)** Univariate Cox analysis of TIME-GES genes in the melanoma cohort of GSE93157. **(G)** Kaplan–Meier survival analysis stratified by TIME-GES score in the melanoma samples from GSE93157.

To assess the predictive capability of TIME-GES in distinguishing tumor immune phenotypes, we utilized the TIME-GES score to generate ROC curves. The analysis showed that the AUC was greater than 0.8, demonstrating strong predictive performance (**Figure 3A**). Additionally, the precision and recall of TIME-GES were 0.7 and 0.9, respectively (**Figure 3B**), with an overall accuracy of 71% in classifying tumors as “cold” and “hot” (**Figure 3C**). To further evaluate the potential of TIME-GES genes as predictors of tumor immune responsiveness, we compared its predictive power against six established tumor immune-related gene signatures (25–30), and four widely used ICB response biomarkers (15). The results showed that TIME-GES outperformed existing signatures in identifying “hot” tumors (**Figure 3D**).

To determine whether TIME-GES could predict tumor response to immunotherapy, we analyzed four independent datasets from patients who had received prior treatments: GSE181815 (31), GSE210287 (32), GSE91061 (33) and GSE93157 (34). Differential expression analysis revealed significantly higher expression for TIME-GES genes in the treatment response group compared to non-responders (**Figure 3E**). In the GSE93157 (34) dataset, which includes PFS data, univariate Cox regression analysis indicated that high TIME-GES gene expression was significantly associated with prolonged PFS (**Figure 3F**). Furthermore, patients with high TIME-GES scores had significantly better PFS outcomes than those with low TIME-GES scores (**Figure 3G**). These findings collectively support the utility of TIME-GES as a biomarker for distinguishing tumor immune phenotypes and predicting tumor immune responses.

### 3.4 TIME-GES is an independent prognostic biomarker

**Figure 4:**
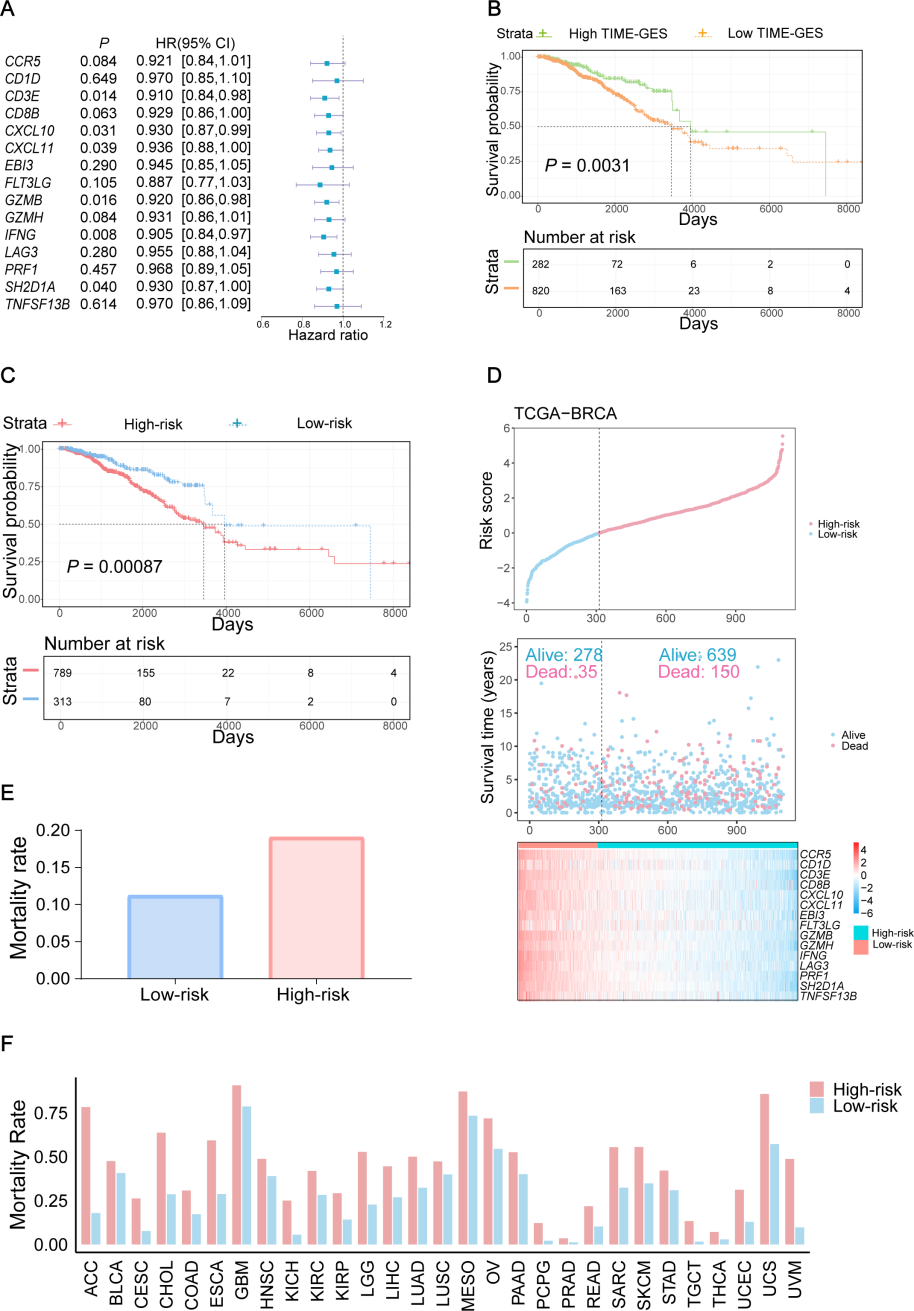
TIME-GES is an independent prognostic biomarker. **(A)** The forest plot showing the result of univariate Cox analysis for TIME-GES genes and OS in BC. **(B, C)** Kaplan–Meier survival curves based on TIME-GES score and risk score in BC. The patients were divided into the high TIME-GES and low TIME-GES groups based on the TIME-GES score, as well as the high-risk low and risk groups due to risk scores. **(D)** Top: Risk scores derived from TIME-GES gene expression and coefficients, ranked across 1102 BC patients. Middle: Distribution of overall survival corresponding to the ranked risk scores. Bottom: Expression heatmap of individual TIME-GES genes in the 1102 patients. **(E)** Correlation between risk scores, and mortality in BC. **(F)** Correlation of risk score with patient mortality across 29 cancer types.

To elucidate the prognostic significance of TIME-GES genes in BC, we calculated the correlation between individual TIME-GES genes and overall survival (OS). The results revealed that high expression of 12 out of 15 genes (80%) was significantly (*P* < 0.05) benefited to prolonged OS (**Supplementary Figure S9**). Univariate Cox proportional hazards regression (HR) further showed the protective role of TIME-GES, with all genes exhibiting HRs below 1.0, indicating a survival benefit (**Figure 4A**). To further evaluate the prognostic usability of the TIME-GES, we derived a composite TIME-GES score and a corresponding risk score. And found that BC patients with high TIME-GES scores exhibited significantly improved OS, PFI, DSS, and DFI compared to those with low scores (**Figure 4B**; **Supplementary Figures S10A–C**). Conversely, patients with low-risk scores displayed superior survival across all measured prognostic indicators (OS, PFI, DSS, DFI) (**Figure 4C**; **Supplementary Figures S10D–F**). This trend was consistently observed across 29 additional cancer types (**Supplementary Figures S11**-**S14**). Furthermore, BC patients with high-risk scores had significantly higher mortality rates compared to those in the low-risk group (**Figures 4D, E**). Cross-cancer analyses further confirmed this pattern, indicating that in the remaining 29 tumor types, patients with high-risk scores exhibited significantly worse survival outcomes (**Figure 4F**). These findings highlight the potential of TIME-GES as a prognostic biomarker and underscore its broader applicability across diverse malignancies.

### 3.5 TIME-GES guided identification of NCD as an immunotherapeutic candidate for TNBC

**Figure 5:**
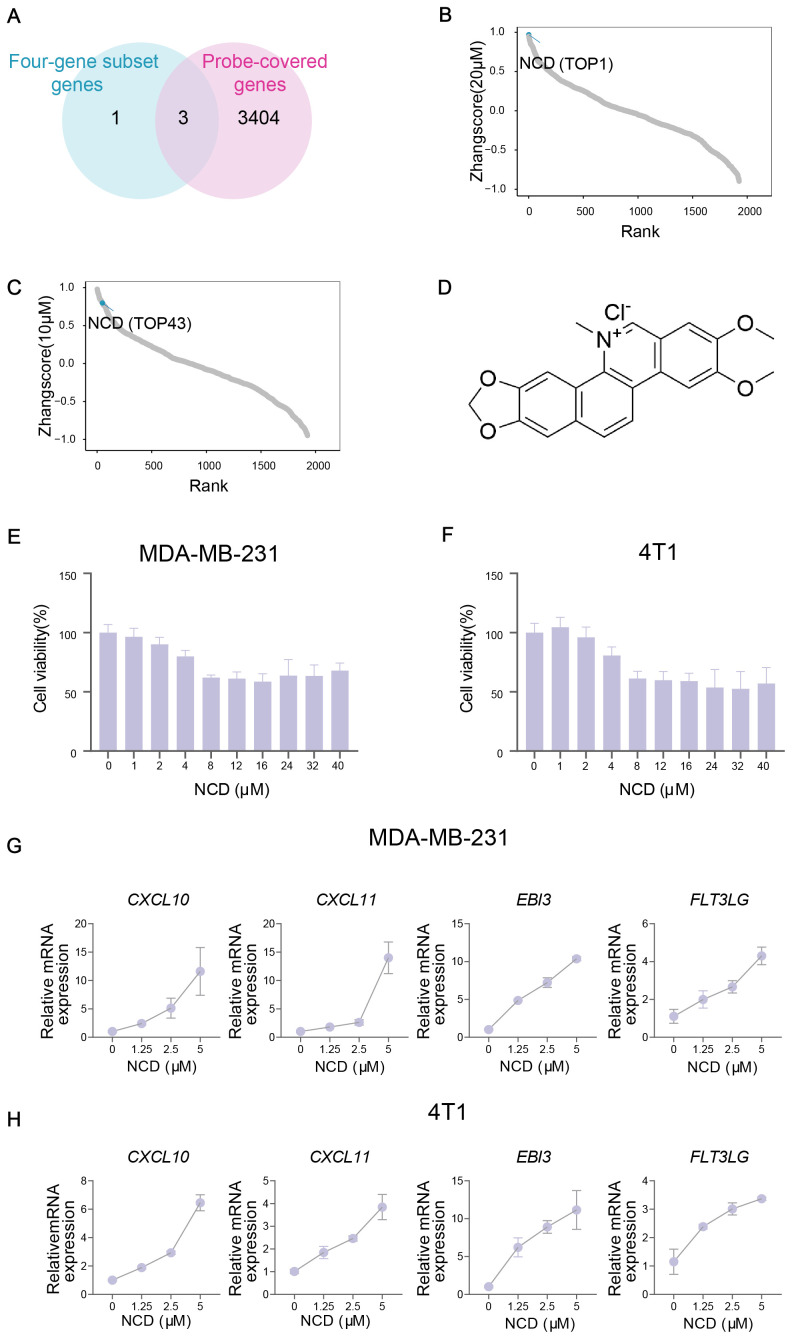
TIME-GES guided identification of NCD as an immunotherapeutic candidate for TNBC. **(A)** Venn diagram showing the overlap between 4 highly expressed genes in TNBC from TIME-GES genes and 3,407 genes detected in large-scale transcriptomic dataset generated from 1,865 natural products perturbation. **(B, C)** The rank of scores for 1,865 natural products at 20 μM and 10 μM concentrations datasets, respectively. **(D)** Chemical structure of NCD. **(E, F)** Cell viability assays in MDA-MB-231 and 4T1 cells after 24 h NCD treatment, respectively (n=3). **(G, H)** mRNA expression of 4 highly expressed genes in BC after NCD treatment in MDA-MB-231 and 4T1 cells, respectively (n=3).

Given the strong associations between the TIME-GES and key tumor immune features, we assessed the utility of TIME-GES for drug screening for TNBC immunotherapy. Firstly, we analyzed the expression profiles of the 15 TIME-GES genes in TNBC samples and identified four genes that were consistently highly expressed in TNBC, hereafter referred to as the four-gene subset (*CXCL10*, *CXCL11*, *EBI3* and *FLT3LG*). To ensure compatibility with the compound screening platform, we next intersected these highly expressed genes with 3,407 genes detected in large-scale transcriptomic dataset generated from 1,865 natural compounds perturbation, yielding three genes (*CXCL11*, *EBI3* and *FLT3LG*) for compound scoring (**Figure 5A**).

Using the three-gene set, we performed Zhangscore analysis across the natural compound library and identified NCD as a promising candidate, ranking 1st at 20μM and 43rd at 10μM, indicating strong and concentration-dependent modulation of TIME-GES expression (**Figures 5B–D**). Then, we assessed its cytotoxicity for MDA-MB-231 and 4T1 TNBC cell lines through CCK-8 and found that NCD exhibited moderate cytotoxicity in both cell lines (**Figures 5E, F**). Moreover, our qPCR results showed that treatment with NCD induced dynamic upregulation of the mRNA expression of the genes, including *CXCL10*, *CXCL11*, *EBI3* and *FLT3LG* (**Figures 5G, H**). These findings highlight NCD as a potential immunomodulatory agent for TNBC, capable of modulating key TIME-GES components and warranting further investigation in preclinical models.

### 3.6 NCD reprograms TIME and suppresses TNBC tumor growth *in vivo*

**Figure 6:**
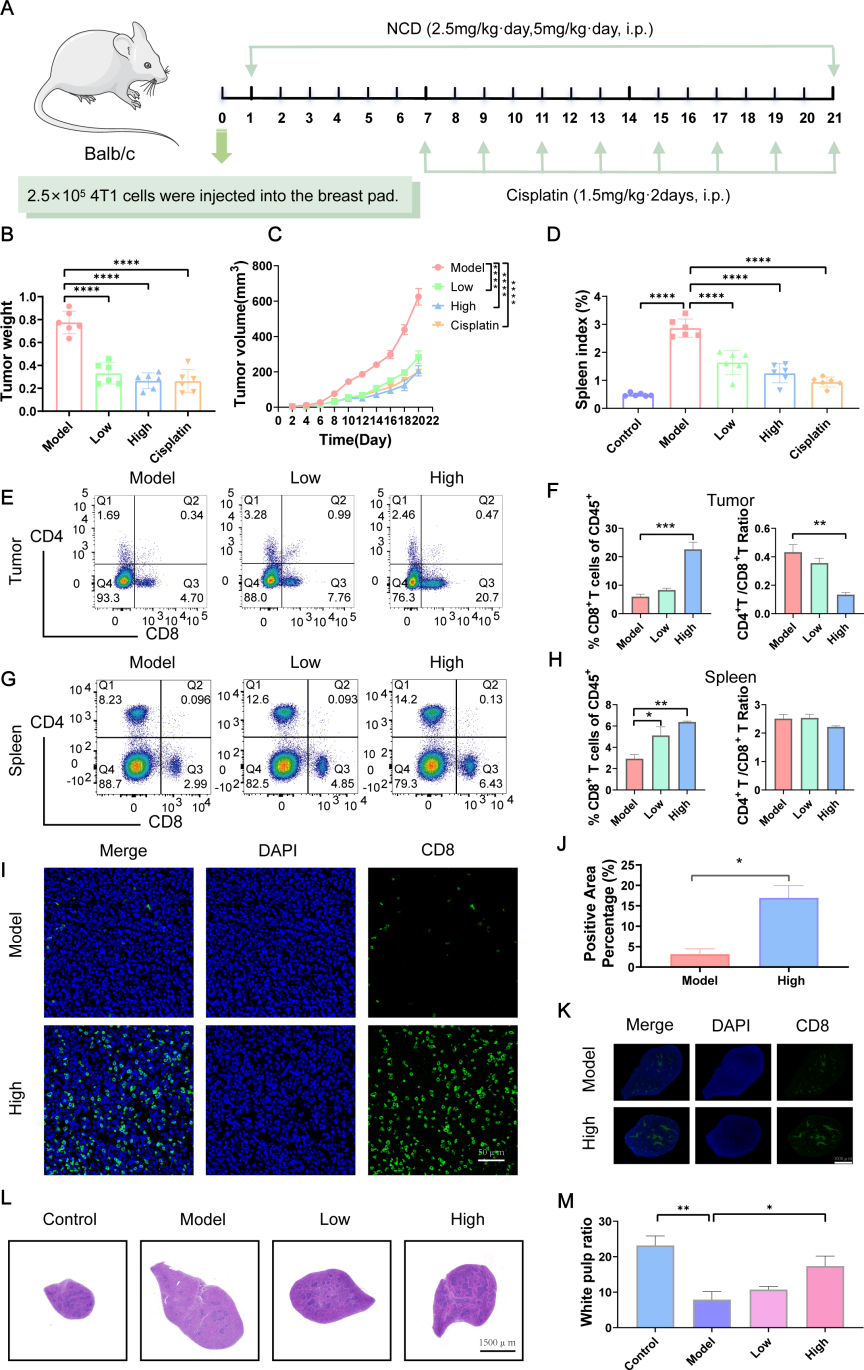
NCD reprograms TIME and suppresses TNBC tumor growth *in vivo*. **(A)** Design of animal experiments. **(B)** Final tumor weight (n=6). **(C)** Tumor growth curves (n=6). **(D)** Mouse spleen index (n=6). **(E)** FCM analysis of CD8^+^ T cells populations in tumor (n=3). **(F)** Quantification of CD8^+^ T cells populations in tumors corresponding to **(E)** (n=3). **(G)** FCM analysis of CD8^+^ T cells populations in spleen (n=3). **(H)** Quantification of CD8^+^ T cells populations in spleen corresponding to **(G)** (n=3). **(I)** IF staining of CD8^+^ T cells in tumors from model and high-dose groups (n=3). **(J)** Quantification of tumor CD8^+^ T IF staining (n=3). **(K)** CD8^+^ T cells IF staining in spleens of model and high-dose groups (n=3). **(L)** H&E staining of mouse spleen (n=3). **(M)** Quantification of spleen H&E staining corresponding to **(L)** (n=3). In all panels, “Control” represents non-tumor-bearing mice, while “Model” indicates mice subjected to tumor induction. Treatment groups are labeled according to NCD dose: “Low” corresponds to 2.5 mg/kg, and “High” to 5 mg/kg. Cisplatin serves as the positive control. *P < 0.05, **P < 0.01, ***P < 0.001.

To investigate the therapeutic efficiency of NCD and its impact on CD8^+^ T cell infiltration in TNBC, we established a tumor-bearing mouse model (**Figure 6A**). Our results showed that NCD treatment significantly inhibited tumor volume and weight, with the high-dose group exhibiting a more pronounced tumor suppression (**Figures 6B, C**; **Supplementary Figure S15A**). Flow Cytometry (FCM) analysis of tumor tissues revealed a significant increase in the proportion of CD8^+^ T cells following NCD treatment, with a dose-dependent enhancement observed across treatment groups. Correspondingly, the CD4^+^/CD8^+^ ratio showed a significant dose-dependent decline (**Figures 6E, F**; **Supplementary Figure S15B**). In the spleen, tumor-bearing mice exhibited splenomegaly and an elevated spleen index; however, the spleen index gradually decreased in a dose-dependent manner with increasing NCD dosage (**Figure 6D**; **Supplementary Figure S15C**). FCM analysis of splenic immune cells similarly showed a dose-dependent increase in the proportion of CD8^+^ T cells and a concurrent decrease in the CD4^+^/CD8^+^ ratio (**Figures 6G, H**; **Supplementary Figure S15B**).

IF staining further validated the increase in CD8^+^ T cell infiltration, demonstrating significantly higher CD8^+^ T cell levels in both tumor and spleen tissues in NCD 5mg/kg (high dose) group compared to the untreated model group (**Figures 6I–K**). Histological examination via H&E staining revealed a significant decrease in white pulp areas in the spleen tumor induction, which gradually recovered in a dose-dependent manner following NCD treatment (**Figures 6L, M**).

To assess the safety profile of NCD, we monitored mouse body weight and organ indices. In NCD 5mg/kg (high dose) group, body weight did not increase over time as observed in the control and NCD 2.5mg/kg (low dose) groups; however, the final weight (15.83 +/- 0.58 g) remained close to the initial value (16.58 +/- 0.14 g), indicating minimal weight fluctuation (**Supplementary Figure S15D**). No significant changes were detected in heart, liver, or lung indices across treatment groups. A modest increase in kidney index was observed at NCD 5mg/kg (high dose) (**Supplementary Figure S15E**), but this was not accompanied by any abnormalities in serum biochemical parameters (AST, ALT, ALB, CREA, UREA), suggesting preserved renal and hepatic function (**Supplementary Figure S15F**). Together, the findings indicate that NCD effectively reshapes the TIME and inhibits TNBC tumor growth *in vivo*, with minimal toxicity, highlighting its potential as a promising immunomodulatory agent for TNBC therapy.

### 3.7 The immune microenvironment–reprogramming function of NCD is mediated by JAK2 inhibition

**Figure 7:**
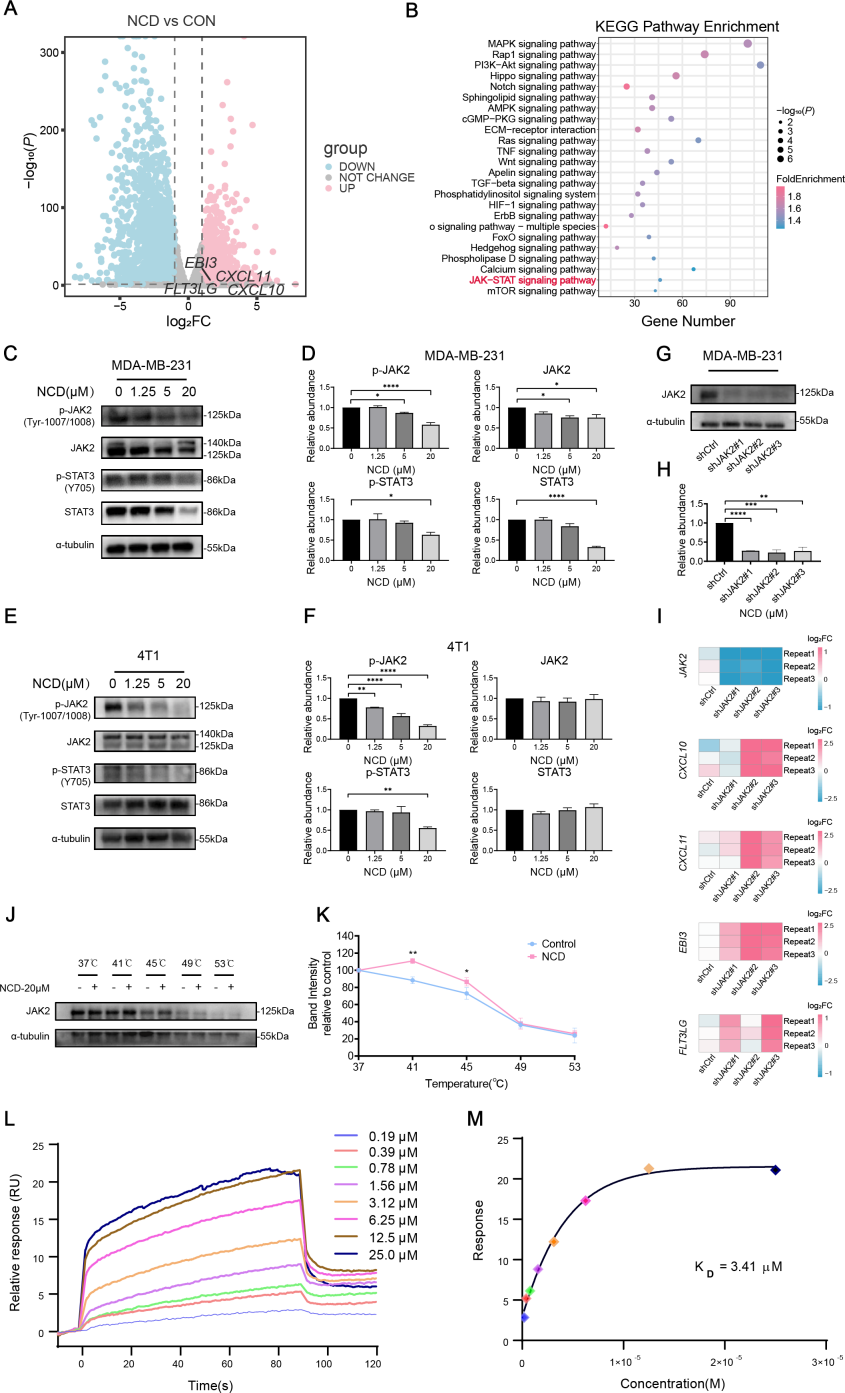
The immune microenvironment–reprogramming function of NCD is mediated by JAK2 inhibition. **(A)** Volcano plot of DEGs in MDA-MB-231 cells treated with or without 5 μM NCD. **(B)** KEGG enrichment analysis of DEGs. **(C)** Western blot of JAK2, p-JAK2, STAT3, and p-STAT3 in MDA-MB-231 cells treated with increasing doses of NCD (n=3). **(D)** Quantification of JAK2, p-JAK2, STAT3, and p-STAT3 proteins corresponding to **(C)** (n = 3). **(E)** Western blot of JAK2, p-JAK2, STAT3, and p-STAT3 in 4T1 cells treated with increasing doses of NCD (n=3). **(F)** Quantification of JAK2, p-JAK2, STAT3, and p-STAT3 proteins corresponding to **(E)** (n = 3). **(G)** Protein expression levels of JAK2 after knockdown of *JAK2* in MDA-MB-231 cell line (n=3). **(H)** Quantification of JAK2 protein levels corresponding to **(G)** (n=3). **(I)** mRNA expression levels of *JAK2*, *CXCL10*, *CXCL11*, *EBI3*, *FLT3LG* after knockdown of *JAK2* in MDA-MB-231 cell (n=3). **(J)** Protein stability of JAK2 under thermal gradient with or without NCD (n=3). **(K)** CETSA Melting Curve (n=3). **(L)** SPR sensorgrams of JAK2 binding to NCD. **(M)** Affinity analysis and KD calculation of JAK2-NCD interaction. *P < 0.05, **P < 0.01, ***P < 0.001, ****P < 0.0001

To elucidate the mechanism by which NCD mediates immune regulation in TNBC, we performed RNA-seq on MDA-MB-231 cells treated with 5 μM NCD. Comparing to negative control DMSO treatment, NCD treatment resulted in the upregulation of *CXCL10*, *CXCL11*, *EBI3* and *FLT3LG* genes (**Figure 7A**). Gene Ontology (GO) enrichment analysis revealed significantly enrichment of immune-related processes, particularly T cell proliferation and activation (**Supplementary Figure S16**) in NCD treated cells. Kyoto encyclopedia of genes and genomes (KEGG) enrichment analysis identified multiple enriched pathways, notably the JAK2-STAT3 signaling pathway, which is known to play a critical role in immune regulation (37) (**Figure 7B**).

To validate the involvement of JAK2-STAT3 signaling pathway, we conducted Western blot experiments in MDA-MB-231 and 4T1 cell lines. In MDA-MB-231 cells, NCD treatment led to a dose-dependent decrease in both total and phosphorylated JAK2 and STAT3 protein (**Figures 7C, D**). In 4T1 cells, phosphorylated JAK2 and STAT3 levels were similarly decreased with increasing NCD dosage, while total protein levels remained unchanged (**Figures 7E, F**). Then, we knocked down the expression of *JAK2* using three different shRNA sequences (**Figures 7G–I**). Our qPCR results showed that JAK2 knockdown upregulated the expression of the four-gene subset (**Figure 7I**), which is further indicated that the regulation of NCD on the expression of the four-gene set is mediated by JAK2 inhibition. To investigate whether NCD directly targets JAK2 protein, we performed CETSA experiment and our results suggested that JAK2 might be a direct target of NCD, showing enhanced thermal stability of JAK2 upon NCD treatment (**Figures 7J, K**). Additionally, SPR analysis confirmed a direct interaction between NCD and JAK2, with a measured KD value of 3.41 μM, supporting the notion that NCD binds JAK2 to modulate its activity (**Figures 7L, M**). Collectively, these results suggest that NCD might exert its immunomodulatory and anti-tumor effects by targeting JAK2 and inhibiting the JAK2-STAT3 signaling pathway, thereby promoting the expression of immune-activating genes in TNBC cells.

## 4 Discussion

In this study, we integrated transcriptomic and survival data from multiple patient cohorts to define a gene expression–based immune phenotype signature, termed TIME-GES. This multifunctional biomarker demonstrated strong potential for distinction of tumor immune phenotypes, prognostic evaluation, immunotherapy response prediction, and high-throughput drug screening. Guided by TIME-GES, we identified and validated a candidate drug, NCD, for the treatment of TNBC, which might target JAK2 in tumor cells to inhibit the JAK2-STAT3 signaling pathway. This inhibition promotes the secretion of immune-modulatory factors CXCL10, CXCL11, EBI3, and FLT3LG by tumor cells, thereby alleviating immunosuppression and enhancing CD8^+^ T cell recruitment into the tumor microenvironment and boosting antitumor immunity.

We systematically identified the TIME-GES. Through a combination of computational analyses and experimental validation, we demonstrated its clinical relevance in the following aspects (1): Multifunctional utility of TIME-GES: TIME-GES can serve as a molecular biomarker for tumor immune status, distinction of tumor immune phenotypes, prognosis evaluation, and prediction of response to immunotherapy, thereby supporting personalized treatment strategies (2); TIME-GES–guided targeted drug discovery: Combining TIME-GES with characteristics at the genetic level of different tumors allows the identification of drugs that promote anti-tumor immunity against different cancers. Compared to other immune-related gene signatures, TIME-GES demonstrates superior performance in predicting tumor immunogenicity, particularly in distinguishing between “cold” and “hot” tumors. Unlike most existing signatures that are developed and validated in a single cancer type, TIME-GES has been systematically evaluated across multiple cancer types, highlighting its broad applicability as a multifunctional biomarker. Notably, the predictive potential of TIME-GES has been further validated through both *in vitro* and *in vivo* experiments, a step rarely achieved by other immune-related signatures. Looking forward, evaluation of TIME-GES across diverse patient populations—considering genetic background, environmental influences, and ethnic diversity—may provide valuable insights into its predictive capacity and further reinforce its potential as a versatile platform for precision immunotherapy. Collectively, these key features underscore the advantages of TIME-GES in both research and potential clinical applications.

Using the TNBC model, we demonstrated the utility of TIME-GES and its four-gene subset in predicting and identifying candidate immunomodulatory compounds. At present, our screening strategy relies on evaluating gene expression changes induced by compounds from large-scale natural product libraries, which offers the advantages of high throughput and cost-effectiveness. Looking ahead, integrating cell-based phenotypic readouts with advanced image analysis, machine learning, and other artificial intelligence approaches—together with the use of diverse cell line models—could substantially enhance the depth, breadth, and precision of such screening platforms (38). In parallel, systematically expanding to larger and more structurally diverse compound libraries will broaden the chemical space explored and increase the likelihood of identifying compounds with robust immune-modulating activity, thereby improving the generalizability and translational potential of TIME-GES–based drug discovery. However, a limitation of this study is that experimental validation was confined to the TNBC context, and the relevance of TIME-GES to other tumor types remains to be determined. Future studies could extend this approach to a broader range of malignancies to facilitate drug screening and immunotherapy development for immunologically “cold” tumors. Moreover, clinical evaluation of TIME-GES across diverse cancers may help establish it as a robust and versatile biomarker for precision immunotherapy and translational applications. Finally, it remains to be explored whether the identified compound, NCD, can exert similar immunomodulatory effects in other immune-cold tumor types.

NCD is the main bioactive ingredient of the traditional Chinese medicine *Zanthoxylum nitidum (Roxb.) DC*. and has the classical effects of activating blood circulation and removing blood stasis, detoxifying and subduing swelling (39). Modern pharmacological studies have demonstrated multiple biological activities of NCD (1): Immunosuppressive effects: by modulating NF-κB and other signaling pathways, NCD exerts anti-inflammatory effects in inflammatory diseases such as osteoarthritis (40), inflammatory bowel disease (41), and sepsis (42) (2); Direct antitumor effects: NCD has demonstrated the ability to inhibit the progression of multiple cancers, including—but not limited to—hepatocellular carcinoma (43–45) and ovarian cancer (46–48). Mechanistically, its antitumor activity is associated with the suppression of epithelial-mesenchymal transition-driven metastasis, induction of apoptosis, and arrest of the cell cycle at the G1/S phase. In our study, we found that NCD significantly modulate the expression of immune-related TIME-GES genes such as *CXCL10*, *CXCL11*, *EBI3*, and *FLT3LG*, thereby enhancing antitumor immune responses and suppressing TNBC progression. Our findings deepen our understanding about the immunological mechanism of action of NCD in tumor suppression in TNBC.

Importantly, we observed that these effects were closely associated with increased infiltration of CD8^+^ T cells, underscoring their central role in mediating the immunomodulatory activity of NCD. While our current work focuses on the regulation of CD8^+^ T cell abundance and upstream molecular pathways, future investigations could further explore functional aspects—such as cytotoxic capacity, persistence, and memory formation—which would yield a more comprehensive understanding of how NCD strengthens CD8^+^ T cell–driven antitumor immunity. Equally noteworthy, within the dosing regimen and treatment duration applied in our study, NCD did not produce any observable toxicity in mice. Although earlier studies have reported safety concerns at substantially higher equivalent doses in normal rats (49, 50), our findings highlight a favorable therapeutic window within the tested range. Looking ahead, expanding research to include in-depth *in vitro* and *in vivo* toxicological and pharmacokinetic evaluations, could further clarify immunological effects and NCD’s safety profile, encompassing its cellular toxicity, systemic tolerance, thereby reinforcing its promise as a candidate for translational immunotherapy.

## 5 Conclusion

In summary, our study demonstrates that TIME-GES is a clinically relevant tool for distinction of tumor immune phenotypes, prognosis evaluation, and prediction of immunotherapy responses. Moreover, TIME-GES offers a strategic framework for identifying novel tumor immune-modulatory compounds. Using this approach, we identified NCD as a previously unrecognized tumor immunotherapeutic agent capable of reprogramming the tumor immune microenvironment, through upregulating the TIME-GES genes, ultimately enhancing antitumor immunity. This work might expand therapeutic options for immunologically “cold” tumors and supports the feasibility of biomarker-guided discovery of small molecules to potentiate cancer immunotherapy.

## Data Availability

The data presented in the study are deposited in the GEO repository, accession number GSE294840.

## Glossary

ACC: Adrenocortical Carcinoma
Act_B: Activated B Cell
Act_CD4: Activated CD4^+^ T Cell
Act_CD8: Activated CD8^+^ T Cell
ALB: Albumin
ALT: Alanine Aminotransferase
AST: Aspartate Aminotransferase
AUC: Area Under the Curve
BC: Breast Cancer
BLCA: Bladder Urothelial Carcinoma
BRCA-Basal: Breast Invasive Carcinoma, Basal-like Subtype
BRCA-Her2: Breast Invasive Carcinoma, HER2-Enriched Subtype
BRCA-LumA: Breast Invasive Carcinoma, Luminal A Subtype
BRCA-LumB: Breast Invasive Carcinoma, Luminal B Subtype
CCK-8: Cell Counting Kit-8
CESC: Cervical Squamous Cell Carcinoma and Endocervical Adenocarcinoma
CHOL: Cholangiocarcinoma
COAD: Colon Adenocarcinoma
CREA: Creatinine
DEGs: Differentially Expressed Genes
DEME: Dulbecco’s Modified Eagle’s Medium
DFI: Disease-Free Interval
DLBC: Lymphoid Neoplasm Diffuse Large B-cell Lymphoma
DMSO: Dimethyl Sulfoxide
DSS: Disease-Specific Survival
ESCA: Esophageal Carcinoma
FBS: Fetal Bovine Serum
FCM: Flow Cytometry
GBM: Glioblastoma Multiforme
GO: Gene Ontology
GSEA: Gene Set Enrichment Analysis
H&E: Hematoxylin and Eosin
HiMAP-seq: Highly multiplexed and parallel sequencing
HNSC: Head and Neck Squamous Cell Carcinoma
HNSC-HPV-: Head and Neck Squamous Cell Carcinoma, HPV-Negative
HNSC-HPV+: Head and Neck Squamous Cell Carcinoma, HPV-Positive
HR: Hazard Ratio
HTS^2^: high-throughput sequencing-based high-throughput screening
ICB: Immune Checkpoint Blockade
iDC: Immature Dendritic Cell
IF: Immunofluorescence
Imm_B: Immature B cell
KEGG: Kyoto Encyclopedia of Genes and Genomes
KICH: Kidney Chromophobe
KIRC: Kidney Renal Clear Cell Carcinoma
KIRP: Kidney Renal Papillary Cell Carcinoma
LGG: Brain Lower Grade Glioma
LIHC: Liver Hepatocellular Carcinoma
LUAD: Lung Adenocarcinoma
LUSC: Lung Squamous Cell Carcinoma
MESO: Mesothelioma
NCD: Nitidine Chloride
NK: Natural Killer Cell
NKT: Natural Killer T Cell
OS: Overall Survival
OV: Ovarian Serous Cystadenocarcinoma
PAAD: Pancreatic Adenocarcinoma
PCPG: Pheochromocytoma and Paraganglioma
pDC: Plasmacytoid Dendritic
PFI: Progression-Free Interval
PFS: Progression-Free Survival
PRAD: Prostate Adenocarcinoma
qPCR: Quantitative Polymerase Chain Reaction
READ: Rectum Adenocarcinoma
RNA-seq: RNA sequencing
ROC: Receiver Operating Characteristic
RPMI: Roswell Park Memorial Institute
SARC: Sarcoma
SKCM: Skin Cutaneous Melanoma
SKCM-Metastasis: Skin Cutaneous Melanoma, Metastatic
SKCM-Primary: Skin Cutaneous Melanoma, Primary Tumor
SPR: Surface Plasmon Resonance
STAD: Stomach Adenocarcinoma
Tem_CD4: Effector Memory CD4^+^ T Cell
Tem_CD8: Effector Memory CD8^+^ T Cell
TGCT: Testicular Germ Cell Tumors
Th1: Type 1 T Helper Cell
THCA: Thyroid Carcinoma
THYM: Thymoma
TIL: Tumor-Infiltrating Lymphocyte
TIME: Tumor Immune Microenvironment
TIME-GES: Tumor Immune Microenvironment Gene Expression Signature
TNBC: Triple-Negative Breast Cancer
UCEC: Uterine Corpus Endometrial Carcinoma
UCS: Uterine Carcinosarcoma
UVM: Uveal Melanoma
