# Supplementary material for: A tumor immune microenvironment gene expression signature for predicting prognosis, immunotherapy efficacy, and drug candidates in triple-negative breast cancer
Source: Front Immunol. 2025 Nov 10;16:1676768. doi: 10.3389/fimmu.2025.1676768 (PMC12640804; doi:10.3389/fimmu.2025.1676768)
Supplement: Supplementary file 1 [file Image1.pdf]

# A Tumor Immune Microenvironment Gene Expression Signature for Predicting Prognosis, Immunotherapy Efficacy, and Drug Candidates in Triple-Negative Breast Cancer

Li Bai[1,#], Ziyu Zhou[1,#], Qingqing Yu[2], Zhen Ye[2], Qingzhou Li[2], Shengrong Li[1], Congcong Li[2], Yu Hu[2], Yunjie Hu[1], Xinran Tai[2], Lei Xiang[2], Sijuan Sun[1], Jianya Deng[2], Yumei Wang[2,*], Dong Wang[2,*]

[1] School of Pharmacy, State Key Laboratory of Southwestern Chinese Medicine Resources, Chengdu University of Traditional Chinese Medicine, Chengdu 611137, China.

[2] School of Basic Medical Sciences, Chengdu University of Traditional Chinese Medicine, Chengdu 611137, China.

[#]These authors contribute equally to this work.

[*]Correspondence: Yumei Wang, yumeiwang@cdutcm.edu.cn; Dong Wang, dwang@cdutcm.edu.cn

Abbreviations are defined in the Abbreviations list.

This file includes: Figure S1 to S16 and Table S1 to S2.

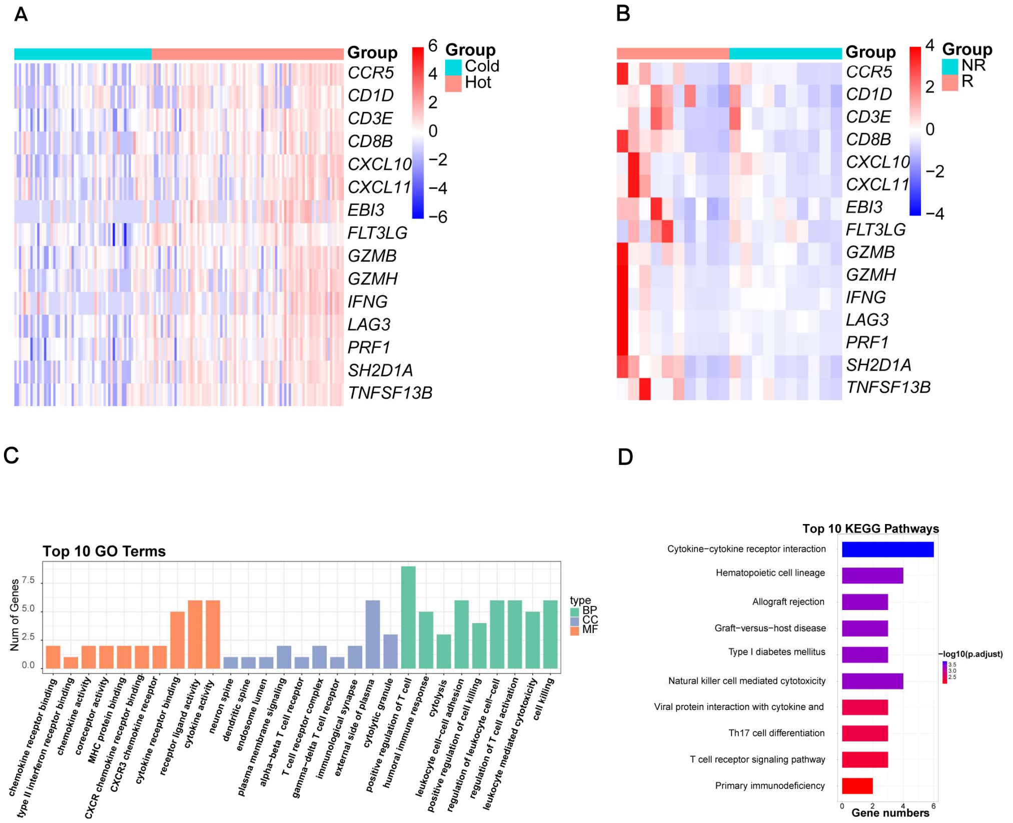


**Fig. S1. The bioinformatics analyses of TIME-GES.** (A, B) Heatmaps of TIME-GES genes expression in lung adenocarcinoma cohort and anti–PD-1-treated melanoma cohort, respectively. (C, D) KEGG and GO enrichment of TIME-GES genes.

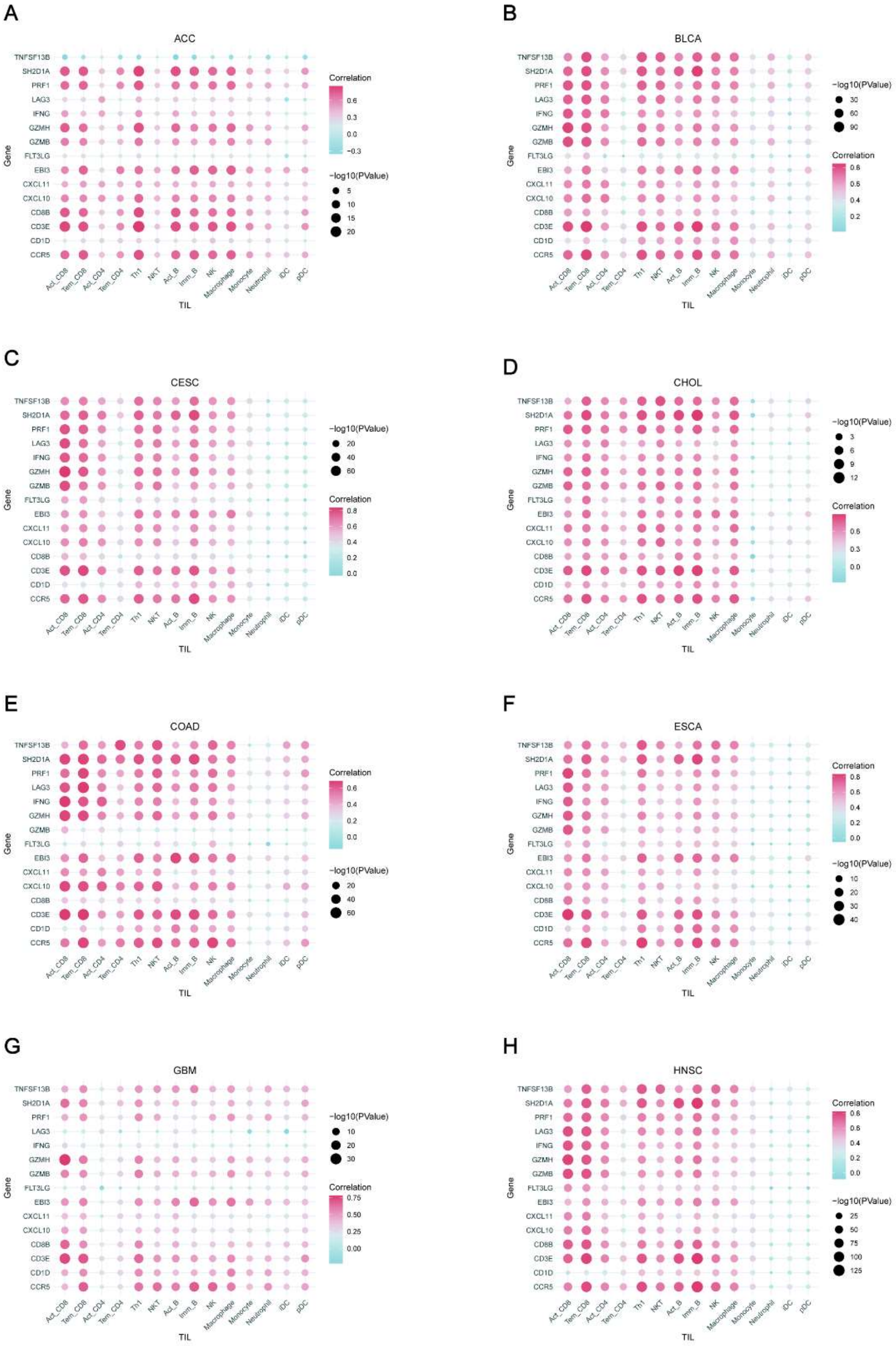


**Fig. S2. The expression of TIME-GES genes correlates with the infiltration of 14 immune cell types in ACC, BLCA, CESC, CHOL, COAD, ESCA, GBM, and HNSC.** (A-H) Correlation of TIME-GES gene expression with 14 immune cell types in ACC (A), BLCA (B), CESC (C), CHOL (D), COAD (E), ESCA (F), GBM (G), and HNSC (H).

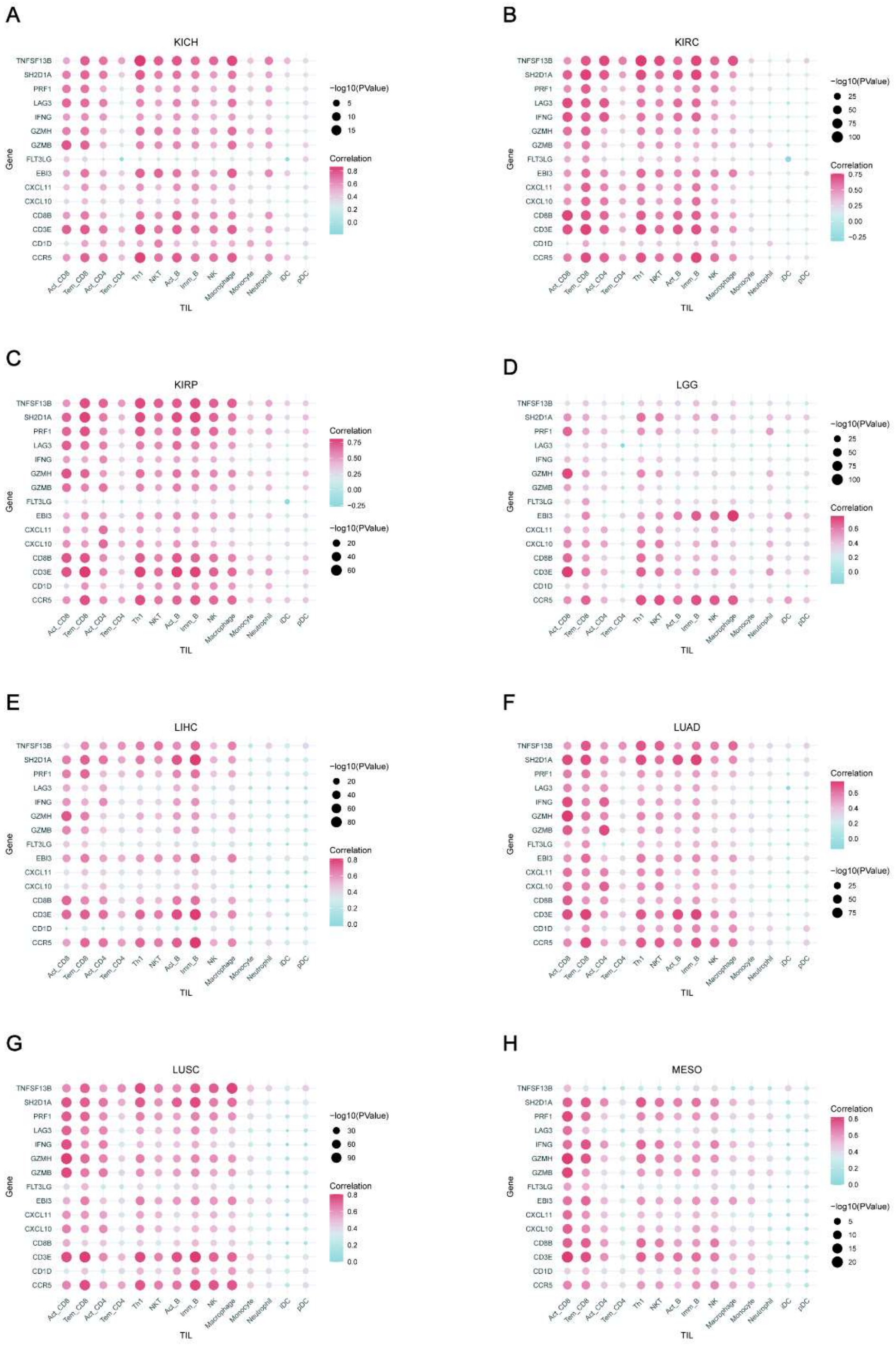


**Fig. S3. The expression of TIME-GES genes correlates with the infiltration of 14 immune cell types in KICH, KIRC, KIRP, LGG, LIHC, LUAD, LUSC, and MESO.** (A-H) Correlation of TIME-GES gene expression with 14 immune cell types in KICH (A), KIRC (B), KIRP (C), LGG (D), LIHC (E), LUAD (F), LUSC (G), and MESO (H).

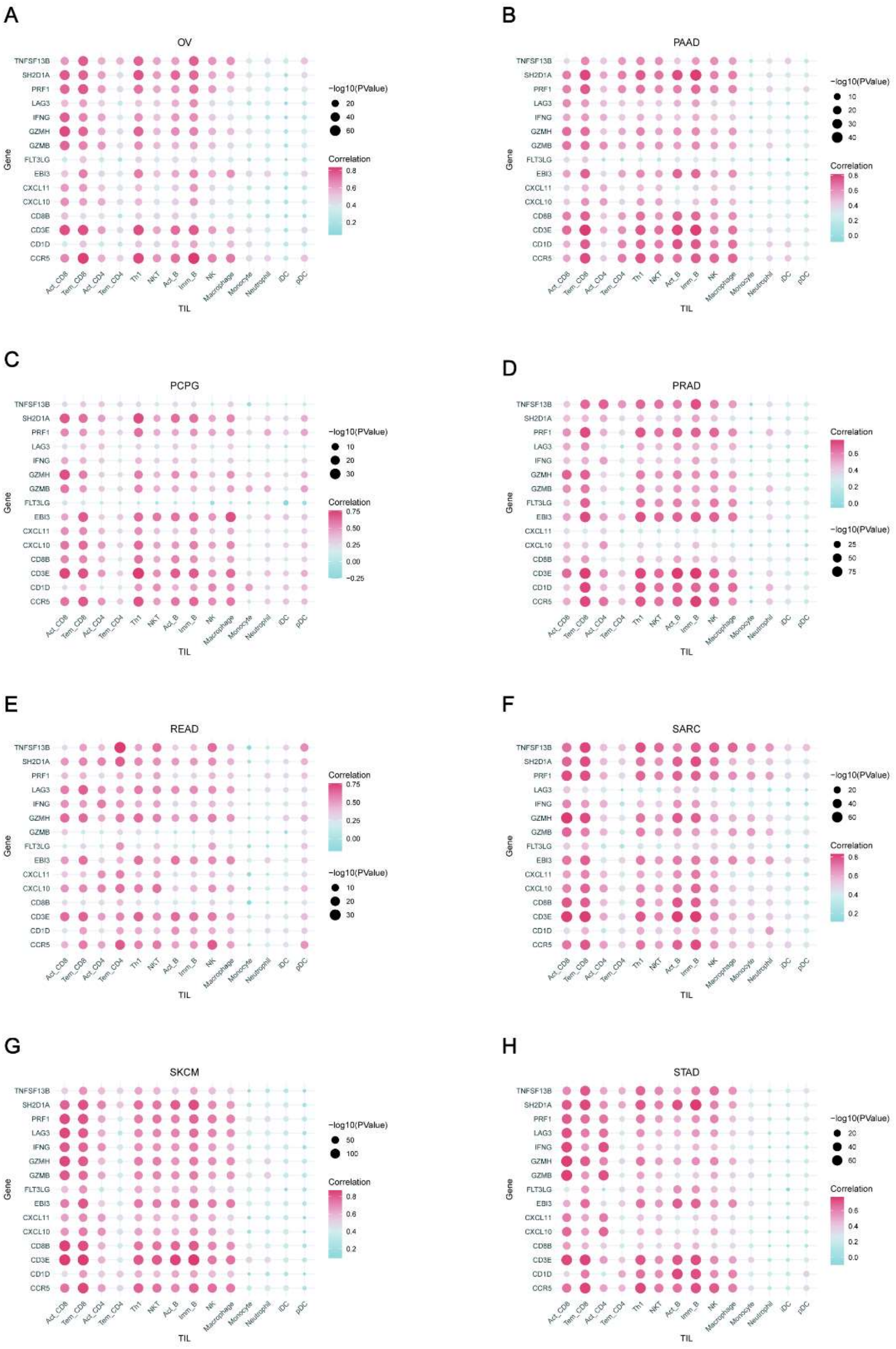


**Fig. S4. The expression of TIME-GES genes correlates with the infiltration of 14 immune cell types in OV, PAAD, PCPG, PRAD, READ, SARC, SKCM, and STAD.** (A-H) Correlation of TIME-GES gene expression with 14 immune cell types in OV (A), PAAD (B), PCPG (C), PRAD (D), READ (E), SARC (F), SKCM (G), and STAD (H).

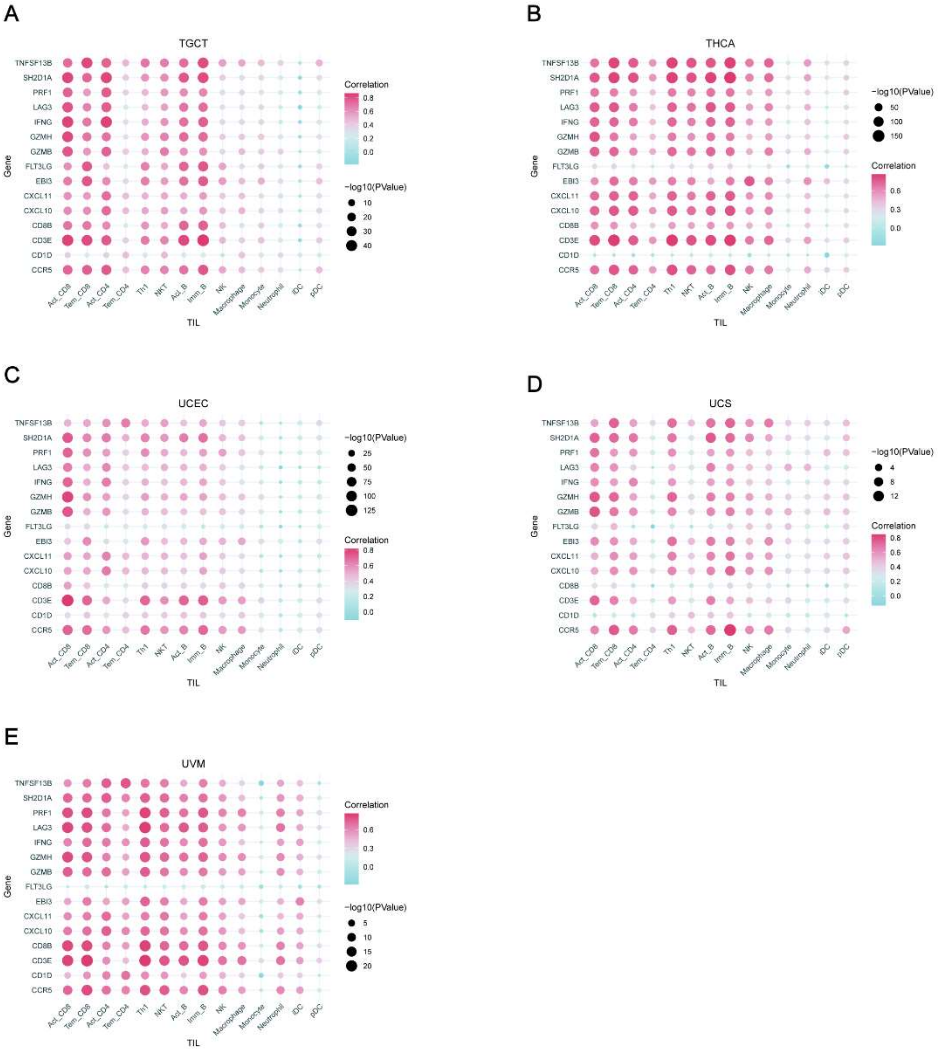


**Fig. S5. The expression of TIME-GES genes correlates with the infiltration of 14 immune cell types in TGCT, THCA, UCEC, UCS, and UVM.** (A-E) Correlation of TIME-GES gene expression with 14 immune cell types in TGCT (A), THCA (B), UCEC (C), UCS (D), and UVM (E).

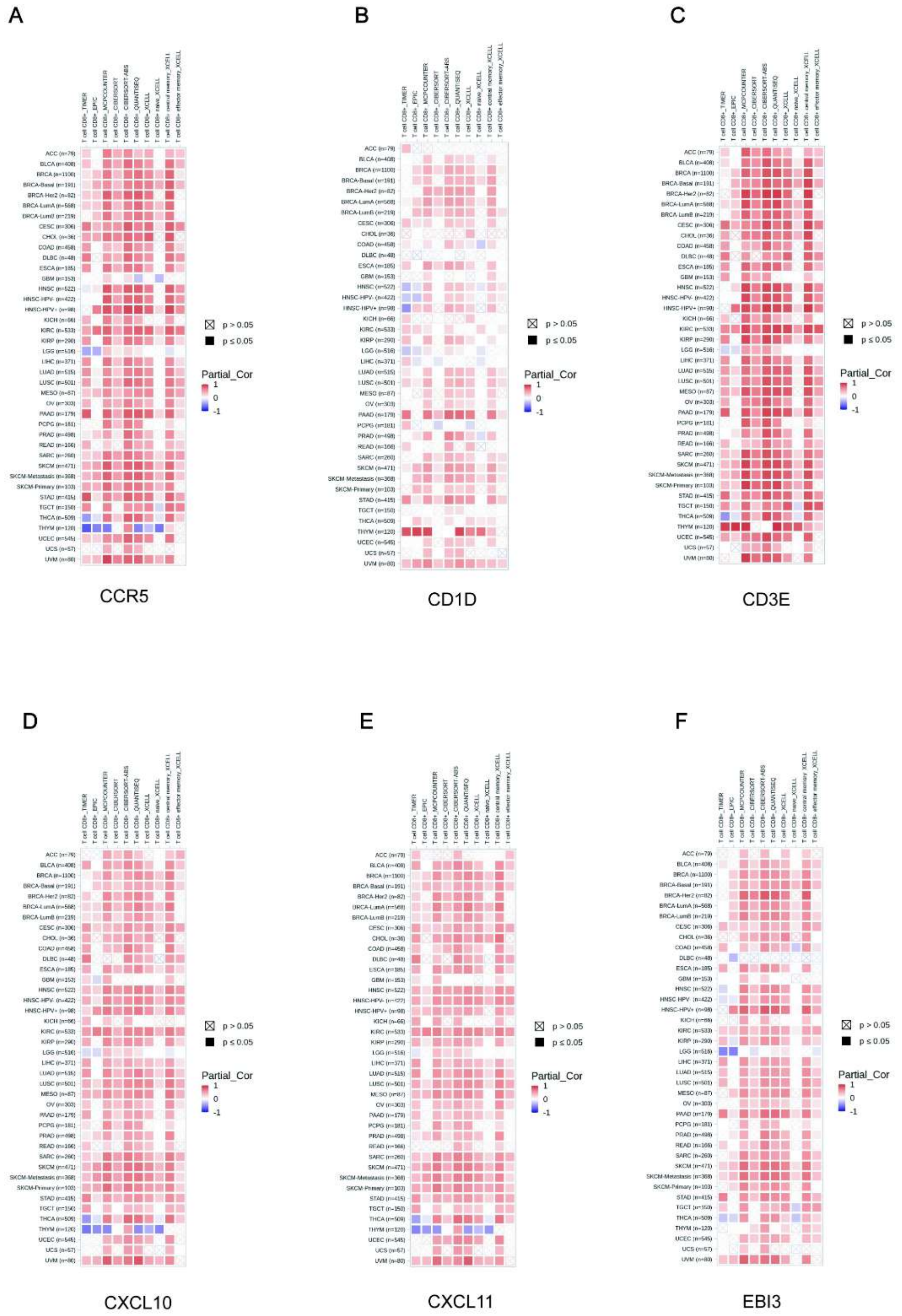


**Fig. S6. The expression of TIME-GES genes (*CCR5*, *CD1D*, *CD3E*, *CXCL10*, *CXCL11*, and *EBI3*) is associated with T cell infiltration in pan-cancer.** (A-F) T cells were associated with the genes *CCR5* (A), *CD1D* (B), *CD3E* (C), *CXCL10* (D), *CXCL11* (E), and *EBI3* (F).

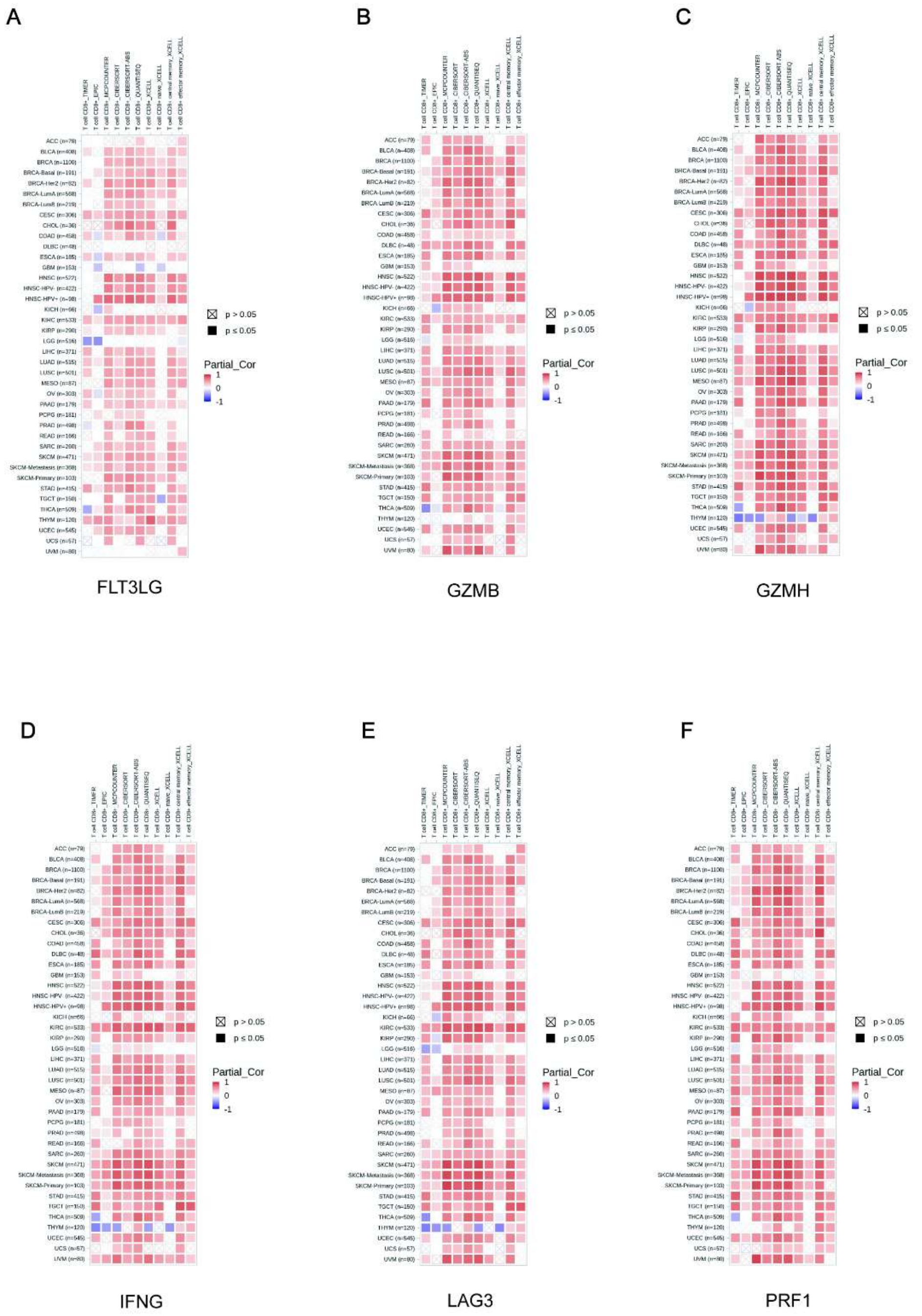


**Fig. S7. The expression of TIME-GES genes (*FLT3LG*, *GZMB*, *GZMH*, *IFNG*, *LAG3*, and *PRF1*) is associated with T cell infiltration in pan-cancer.** (A-F) T cells were associated with the genes *FLT3LG* (A), *GZMB* (B), *GZMH* (C), *IFNG* (D), *LAG3* (E), and *PRF1* (F).

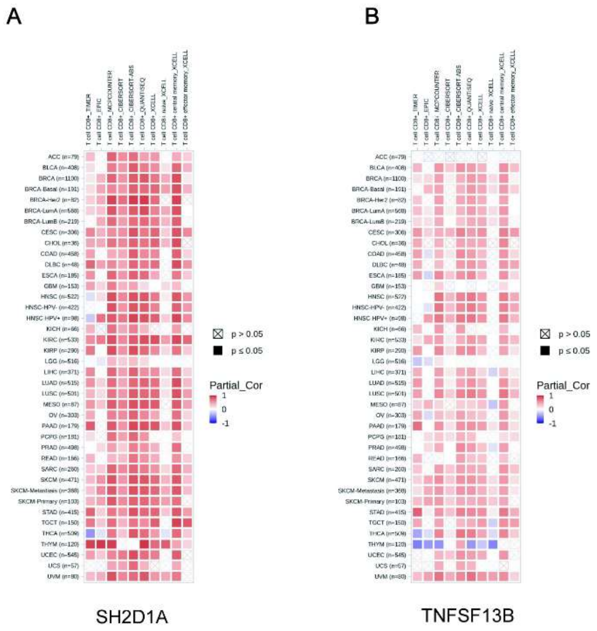


**Fig. S8. The expression of TIME-GES genes (*SH2D1A* and *TNFSF13B*) is associated with T cell infiltration in pan-cancer.** (A, B) T cells were associated with the genes *SH2D1A* (A) and *TNFSF13B* (B).

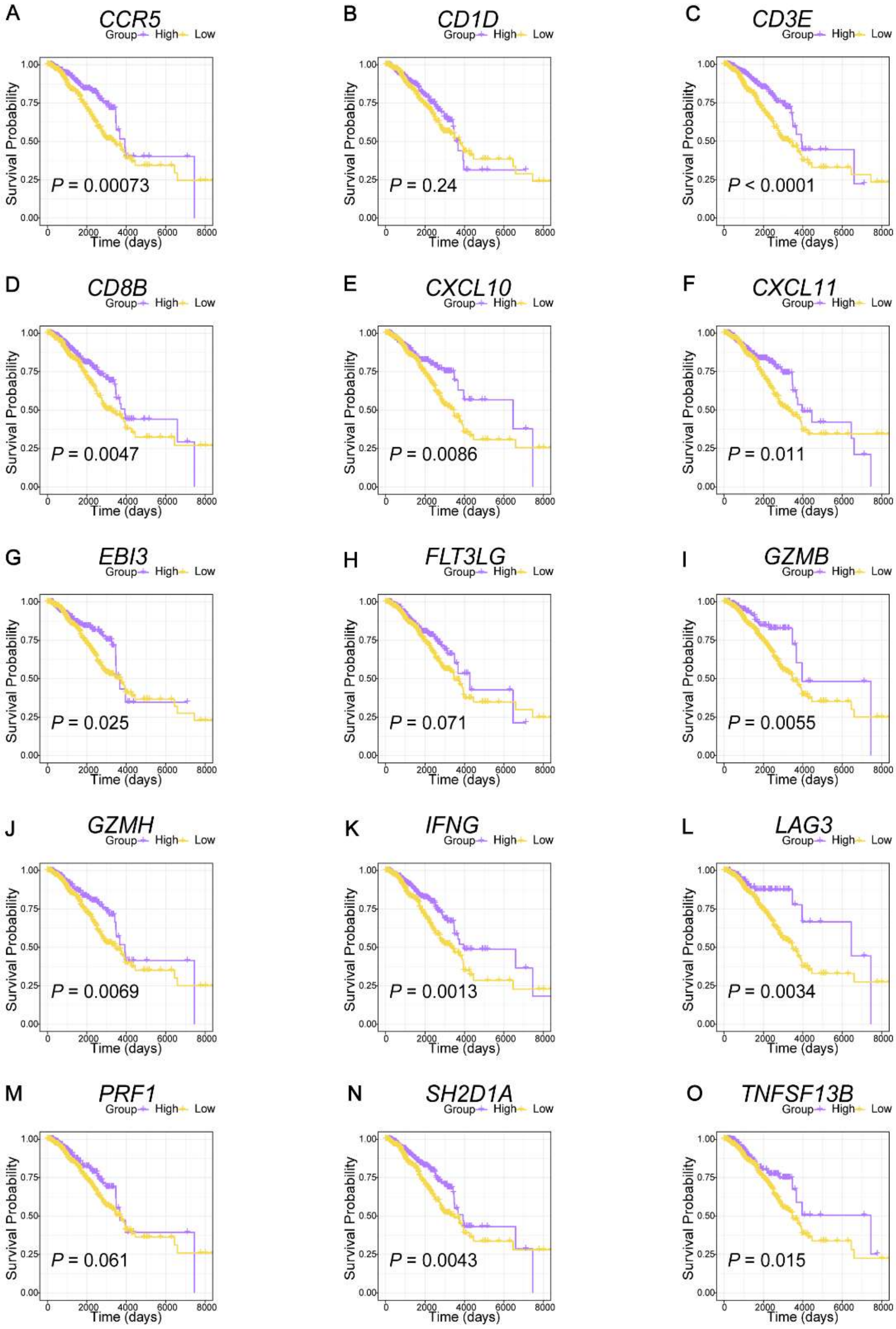


**Fig. S9. TIME-GES genes are associated with prognosis in BC.** (A-O) Kaplan–Meier survival curves of TIME-GES genes in BC.

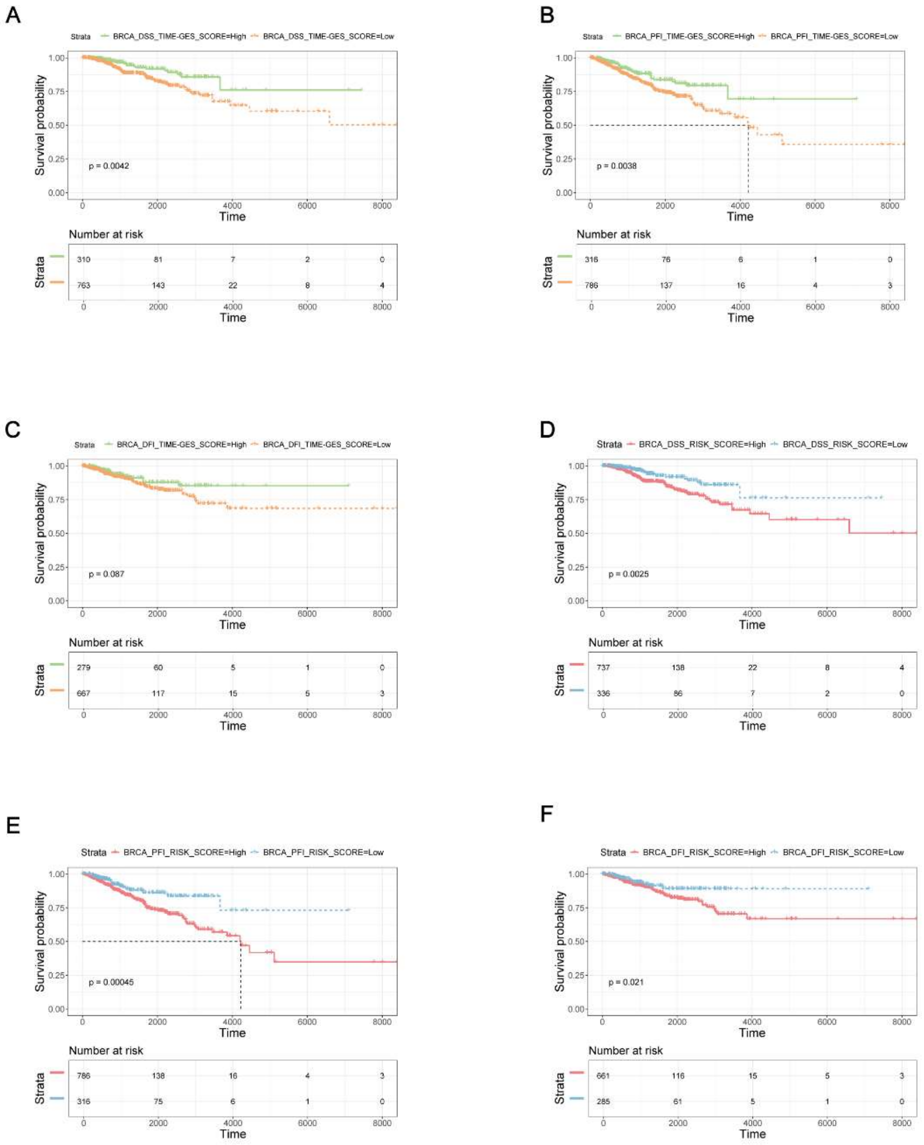


**Fig. S10. TIME-GES as a prognostic biomarker in BC.** (A-C) Kaplan–Meier survival curves for DSS (A), PFI (B), and DFI (C) based on the TIME-GES score. (D-F) Kaplan–Meier survival curves for DSS (D), PFI (E), and DFI (F) based on the risk score. The patients were divided into the high TIME-GES group and the low TIME-GES group due to the TIME-GES score. The patients were divided into the high TIME-GES and low TIME-GES groups, as well as the high-risk low and risk groups due to risk scores.

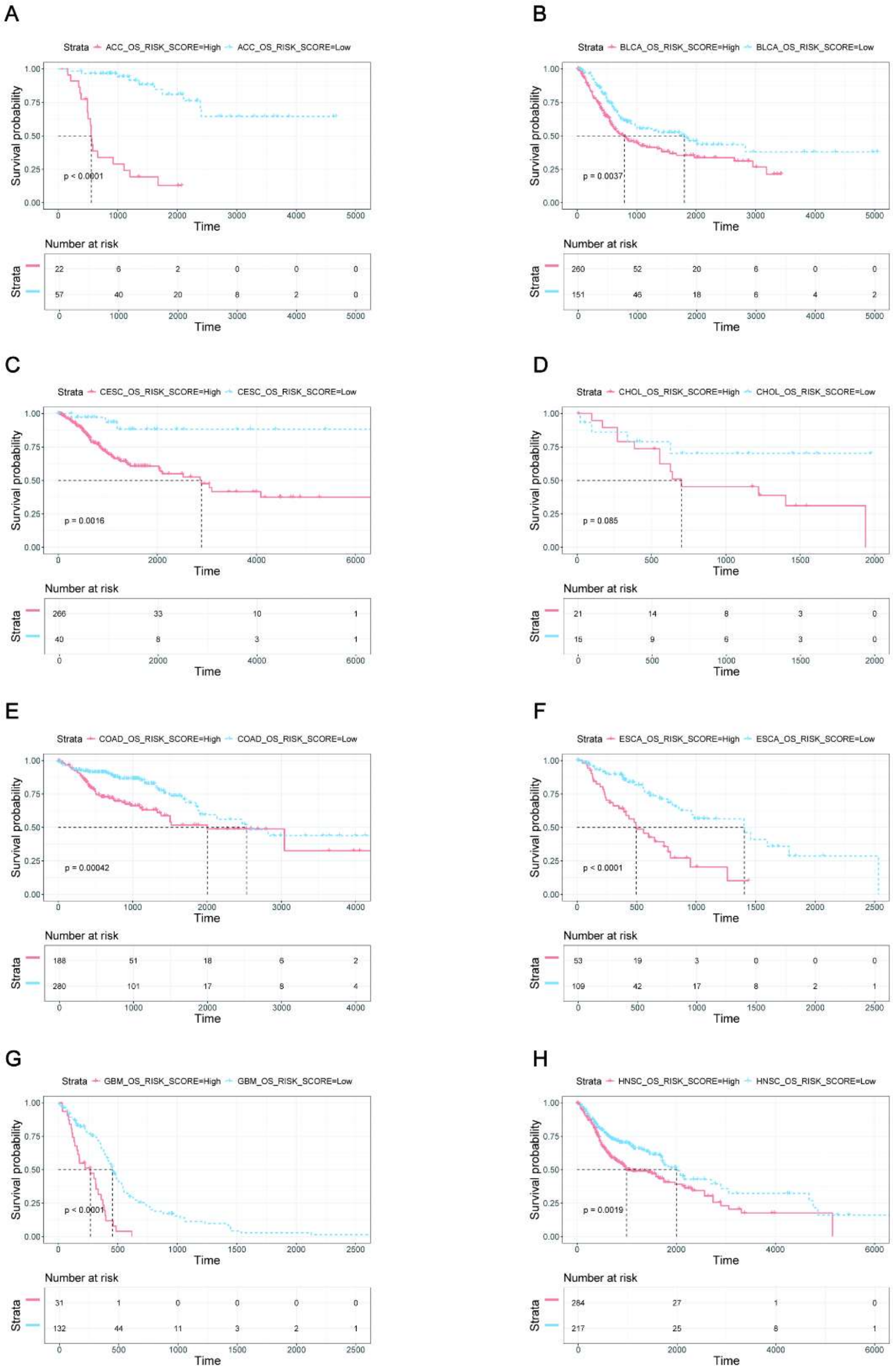


**Fig. S11. TIME-GES as an effective prognostic biomarker in ACC, BLCA, CESC, CHOL, COAD, ESCA, GBM, and HNSC.** (A-H) Kaplan–Meier survival curves for OS based on risk score in ACC (A), BLCA (B), CESC (C), CHOL (D), COAD (E), ESCA (F), GBM (G), and HNSC (H). The patients were divided into the high TIME-GES group and the low TIME-GES group due to the TIME-GES score.

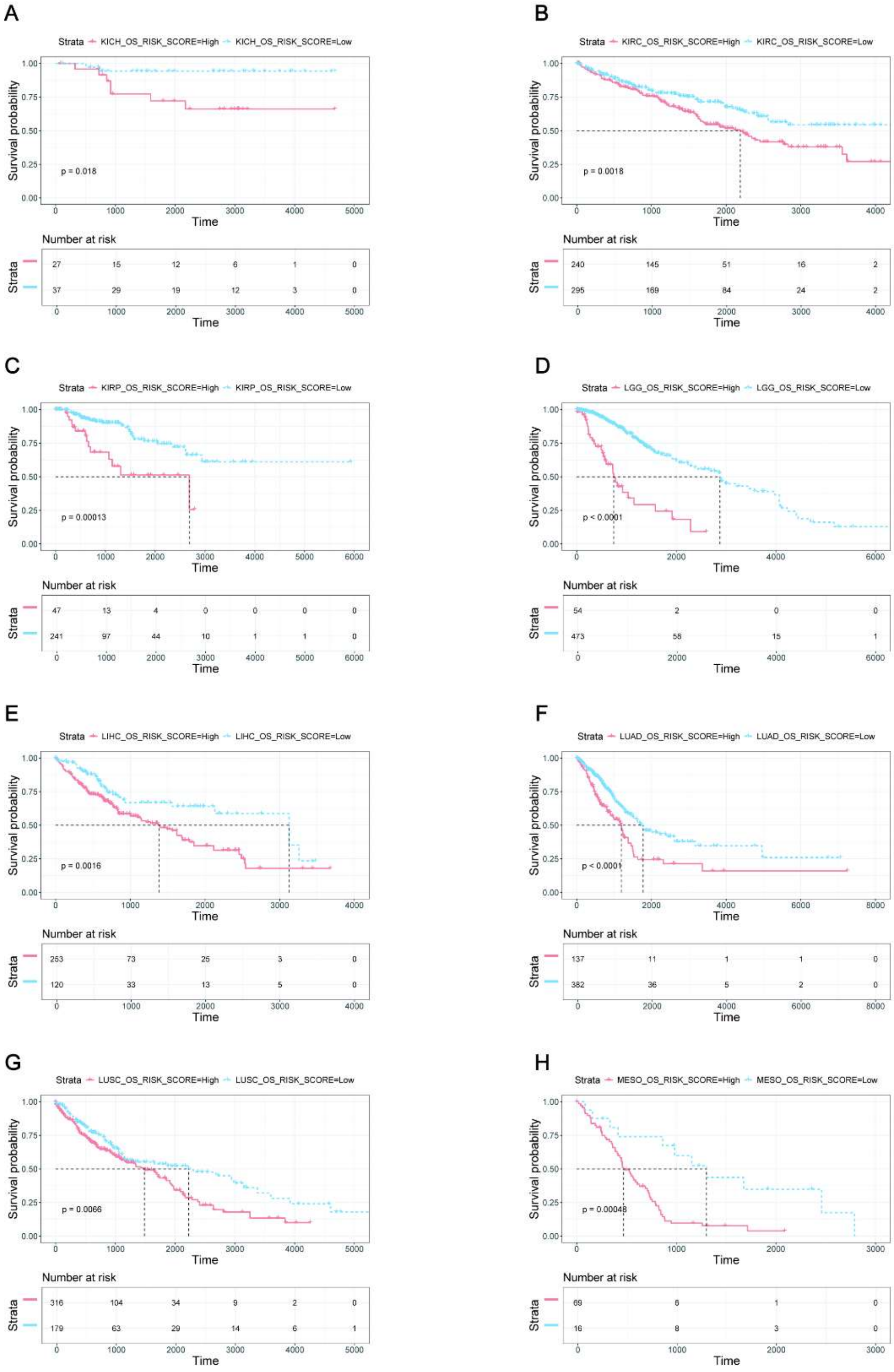


**Fig. S12. TIME-GES as an effective prognostic biomarker in KICH, KIRC, KIRP, LGG, LIHC, LUAD, LUSC, and MESO.** (A-H) Kaplan–Meier survival curves for OS based on risk score in KICH (A), KIRC (B), KIRP (C), LGG (D), LIHC (E), LUAD (F), LUSC (G), and MESO (H). The patients were divided into the high TIME-GES group and the low TIME-GES group due to the TIME-GES score.

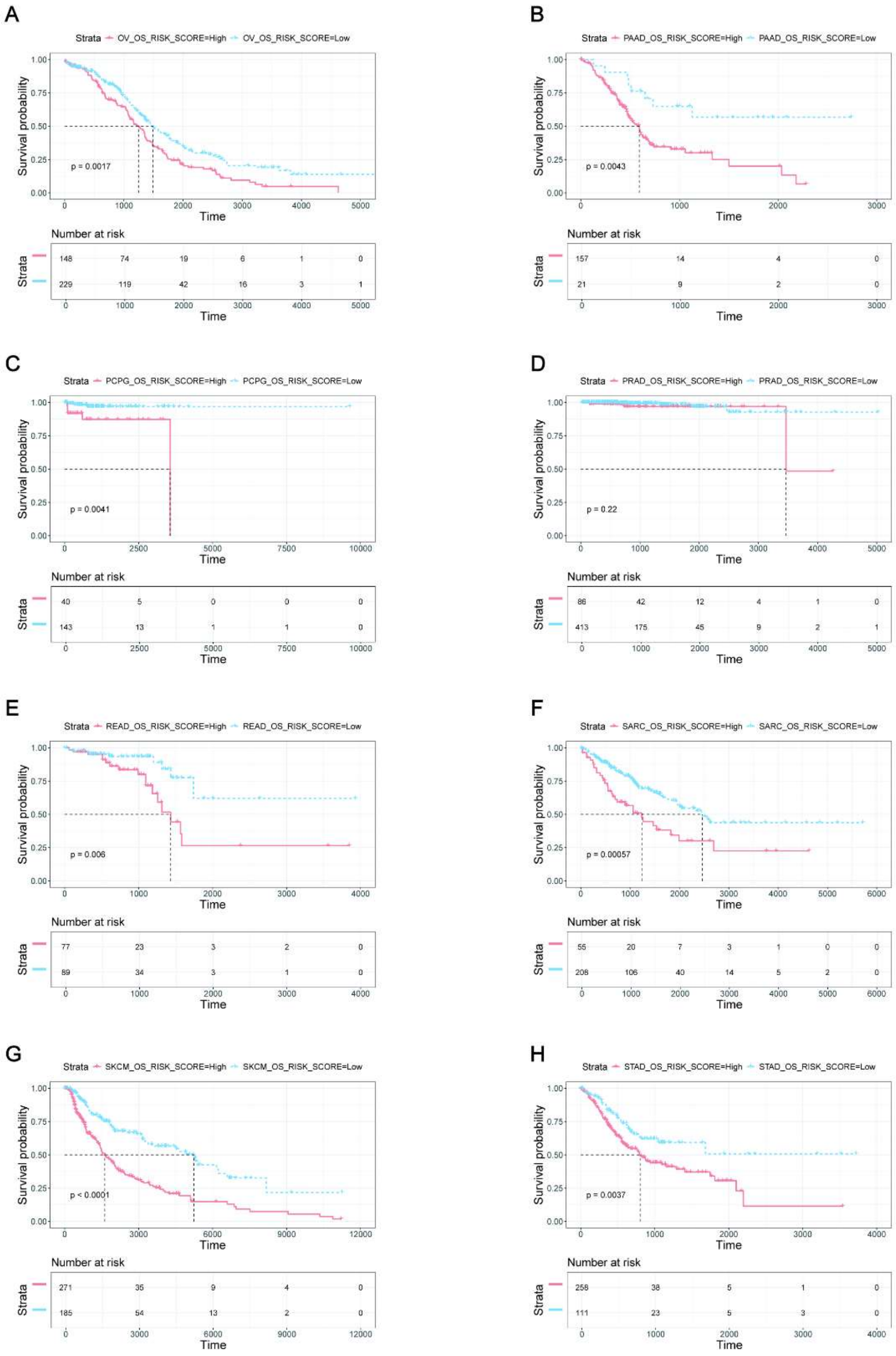


**Fig. S13. TIME-GES as an effective prognostic biomarker in OV, PAAD, PCPG, PRAD, READ, SARC, SKCM, and STAD.** (A-H) Kaplan–Meier survival curves for OS based on risk score in OV (A), PAAD (B), PCPG (C), PRAD (D), READ (E), SARC (F), SKCM (G), and STAD (H). The patients were divided into the high TIME-GES group and the low TIME-GES group due to the TIME-GES score.

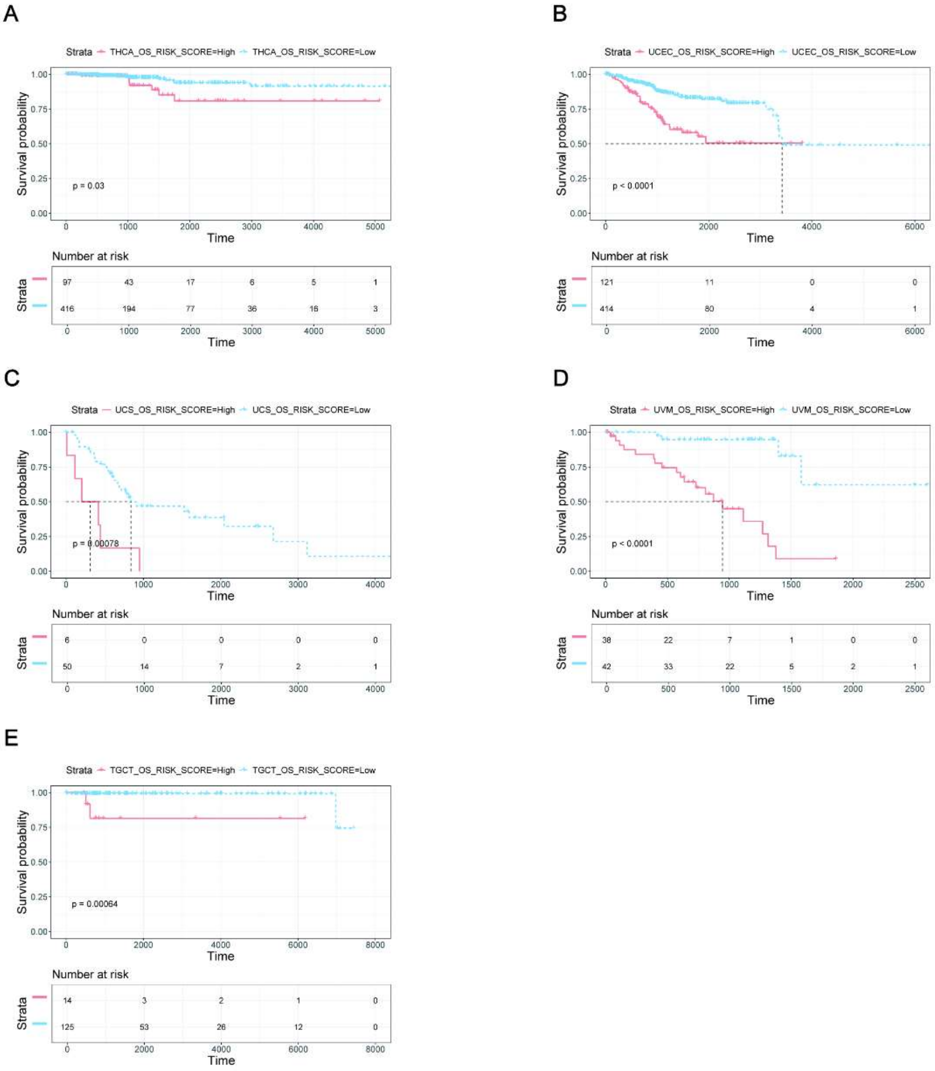


**Fig. S14. TIME-GES as an effective prognostic biomarker in TGCT, THCA, UCEC, UCS, and UVM.** (A-E) Kaplan–Meier survival curves for OS based on risk score in TGCT (A), THCA (B), UCEC (C), UCS (D), and UVM (E). The patients were divided into the high TIME-GES group and the low TIME-GES group due to the TIME-GES score.

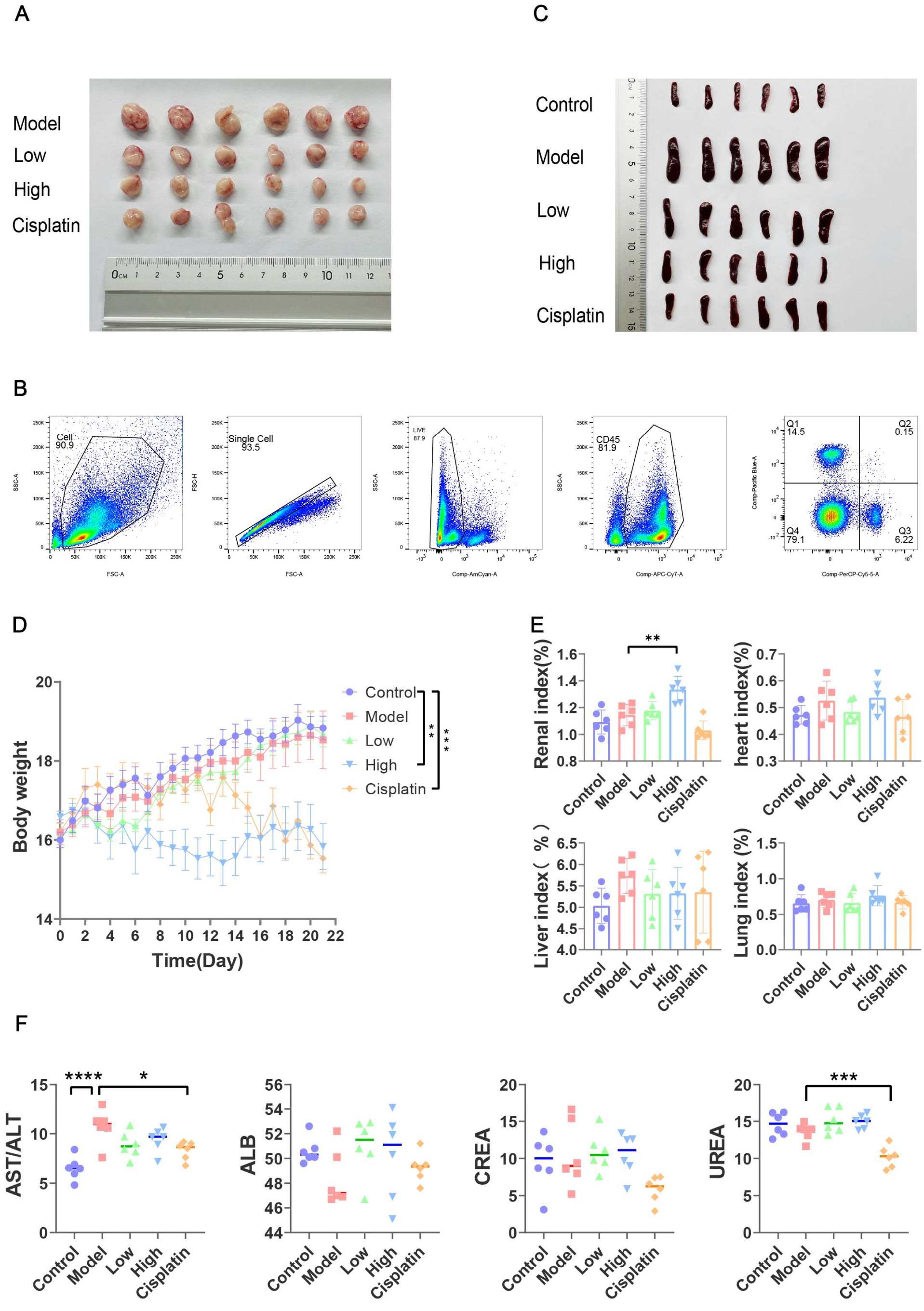


**Fig. S15. NCD exhibits significant therapeutic efficacy in TNBC.** (A) Tumor morphology in tumor-bearing mice. (B) FCM gating strategy for tumor and spleen. (C) Mouse spleen diagram. (D) Body weight of mice. (E) Organ index of kidney, liver, heart, and lung. (F) Serum biochemistry (AST/ALT, ALB, CREA, UREA).

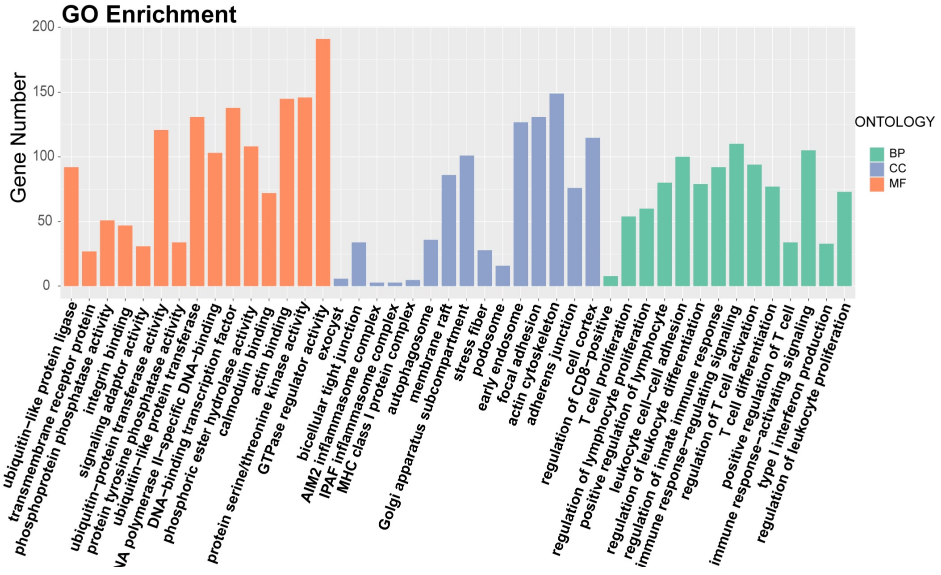


**Fig. S16. GO enrichment analysis of DEGs after NCD treatment.**

**Table S1. The list of primers used for qPCR.**

| Human Gene | Primer | Primer squence | Mouse Gene | Primer | Primer squence |
|---|---|---|---|---|---|
| *CXCL10* | Forward | GGTGAGAAGAGATGTCTGAATCC | *Cxcl10* | Forward | GACCTTTTTTGGCTAAACGCTTTC |
| | Reverse | GTCCATCCTTGGAAGCACTGCA | | Reverse | ATCATCCCTGCGAGCCTATCCT |
| *CXCL11* | Forward | AAGGACAACGATGCCTAAATCCC | *Cxcl11* | Forward | CCTGCATTATGAGGCGAGCTTG |
| | Reverse | CAGATGCCCTTTTCCAGGACTTC | | Reverse | CCGAGTAACGGCTGCGACAAAG |
| *EBI3* | Forward | CTGGATCCGTTACAAGCGTCAG | *Ebi3* | Forward | CTCTCAAGTACCGACTCCGCTA |
| | Reverse | CACTTGGACGTAGTACCTGGCT | | Reverse | CTGAGCTGACACCTGGATGCAA |
| *FLT3LG* | Forward | GCTGGGTCCAAGATGCAAGG | *Flt3lg* | Forward | GGGGGACACCTGACTGTTAC |
| | Reverse | GGTCTGGACGAAGCGAAGAC | | Reverse | TGTTGACGTCCTCCAGAAGC |
| *β-ACTIN* | Forward | CATGAAGTGTGACGTGGACATC | *β-actin* | Forward | TAGGCACCAGGGTGTGATG |
| | Reverse | CAGGAGGAGCAATGATCTTGATC | | Reverse | GTGGTGCCAGATCTTCTCCA |
| *JAK2* | Forward | TCTGGGGAGTATGTTGCAGAA | - | - | - |
| | Reverse | AGACATGGTTGGGTGGATACC | | - | - |

**Table S2. shRNA Information.**

| Name | ID | TRC_ID | CLONE_NAME | OLIG_SEQ |
|---|---|---|---|---|
| Control | shCtrl | - | - | GGTGTATGGGCTACTATAGAA |
| JAK2 | ShJAK2#1 | TRCN0000003178 | NM_004972.x-4564s1c1 | CCGGCCCTGACCCTAAATAATACATCTCGAGATGTATTATTTAGGGTCAGGGTTTTT |
| | ShJAK2#2 | TRCN0000003179 | NM_004972.x-3019s1c1 | CCGGCACAGTTTGAAGAGAGACATTCTCGAGAATGTCTCTCTTCAAACTGTGTTTTT |
| | ShJAK2#3 | TRCN0000003181 | NM_004972.x-2826s1c1 | CCGGGCAGAATTAGCAAACCTTATACTCGAGTATAAGGTTTGCTAATTCTGCTTTTT |
